# Relationship between socioeconomic status and gastrointestinal infections in developed countries: A systematic review and meta-analysis

**DOI:** 10.1371/journal.pone.0191633

**Published:** 2018-01-23

**Authors:** Natalie L. Adams, Tanith C. Rose, Jeremy Hawker, Mara Violato, Sarah J. O’Brien, Benjamin Barr, Victoria J. K. Howard, Margaret Whitehead, Ross Harris, David C. Taylor-Robinson

**Affiliations:** 1 NIHR Health Protection Research Unit in Gastrointestinal Infections, Liverpool, United Kingdom; 2 Department of Public Health and Policy, University of Liverpool, Liverpool, United Kingdom; 3 National Infection Service, Public Health England, London, United Kingdom; 4 Health Economics Research Centre, University of Oxford, Oxford, United Kingdom; Hospital General O'Horan, MEXICO

## Abstract

**Background:**

The association between socioeconomic status (SES) and health is well-documented; however limited evidence on the relationship between SES and gastrointestinal (GI) infections exists, with published studies producing conflicting results. This systematic review aimed to assess the association between SES and GI infection risk, and explore possible sources of heterogeneity in effect estimates reported in the literature.

**Methods:**

MEDLINE, Scopus, Web of Science and grey literature were searched from 1980 to October 2015 for studies reporting an association between GI infections and SES in a representative population sample from a member-country of the Organisation for Economic Co-operation and Development. Harvest plots and meta-regression were used to investigate potential sources of heterogeneity such as age; level of SES variable; GI infection measurement; and predominant mode of transmission. The protocol was registered on PROSPERO: CRD42015027231.

**Results:**

In total, 6021 studies were identified; 102 met the inclusion criteria. Age was identified as the only statistically significant potential effect modifier of the association between SES and GI infection risk. For children, GI infection risk was higher for those of lower SES versus high (RR 1.51, 95% CI;1.26–1.83), but there was no association for adults (RR 0.79, 95% CI;0.58–1.06). In univariate analysis, the increased risk comparing low and high SES groups was significantly higher for pathogens spread by person-to-person transmission, but lower for environmental pathogens, as compared to foodborne pathogens.

**Conclusions:**

Disadvantaged children, but not adults, have greater risk of GI infection compared to their more advantaged counterparts. There was high heterogeneity and many studies were of low quality. More high quality studies are needed to investigate the association between SES and GI infection risk, and future research should stratify analyses by age and pathogen type. Gaining further insight into this relationship will help inform policies to reduce inequalities in GI illness in children.

## Introduction

Gastrointestinal (GI) infections are common. Estimates suggest around 25% of people in the UK suffer an episode of infectious intestinal disease (IID) per year.[1,2] Several risk factors for GI infection have been investigated in the literature, including environmental risk factors such as population density and rurality, and individual-level risk factors such as sex and ethnicity.[3–6] Age has been identified as an important risk factor for GI infection, with the youngest age groups most at risk.[3,7,8] Yet for some potential risk factors such as socioeconomic status (SES), the association has been less clear. Inconsistent results have been observed among studies, with some reporting higher rates of GI infections among lower socioeconomic groups[8–10] and others observing the opposite.[11,12] Lower risk of *Campylobacter*, *Cryptosporidium* and norovirus has been identified in more disadvantaged areas.[5, 13–16] In contrast, incidence was found to be higher in more disadvantaged areas for listeria and rotavirus.[17,18] Disadvantaged children were found to have higher risk of non-typhoidal *Salmonella*, rotavirus and norovirus.[19–21] A systematic review exploring the impact of SES on laboratory confirmed foodborne illness in developed countries identified 16 studies across four pathogens with mixed results, differing by pathogen.[22] These results demonstrate the ongoing disagreements within this area of research.

A systematic review was warranted to summarise and understand the contradictory findings observed in the literature and explore the relationship for GI pathogens which are predominantly transmitted via person-to-person, waterborne and environmental routes as well as the foodborne route which has been studied previously.[22] Our review aimed to explore the relationship between SES and a full range of GI infections to assess the magnitude and direction of the association, and suggest possible explanations for any observed differences.

## Methods

### Search strategy and selection criteria

We conducted a systematic review and meta-analysis. The exposure of interest was SES, measured at the individual or aggregate level by income, education, occupation, employment or area-level deprivation. The primary outcome of interest was incidence/prevalence of any symptomatic GI infection, including syndromic definitions of GI infections without a laboratory diagnosis. These were included as various socioeconomic or healthcare seeking behavioural factors could influence whether an individual is diagnosed with a GI infection.

The methods for this study have been described in detail in the study protocol (https://systematicreviewsjournal.biomedcentral.com/articles/10.1186/s13643-016-0187-7). [23] Full details of the inclusion/exclusion criteria are reported in Table 1.

**Table 1:** Inclusion and exclusion criteria.

Inclusion criteria
1. Studies quantitatively measuring the prevalence or incidence of any symptomatic gastrointestinal infection in a representative population sample
2. Studies quantitatively measuring socioeconomic status at an individual or aggregate level by occupation, income, education, employment or area deprivation
3. Studies reporting a quantitative association between the first two inclusion criteria i.e. reporting an association between gastrointestinal infection and socioeconomic status
4. Studies written or translated into English language
5. Studies reporting on human subjects
6. Subjects selected from the populations of countries that are members of the Organisation for Economic Co-operation and Development (OECD), reporting data after 1980 or the date that they became a member of the OECD
7. Studies reporting on data collected after 1980
8. Observational studies
Exclusion criteria
1. Unrepresentative population sample
2. Outbreak reports
3. Studies analysing travel related cases only
4. Review studies
5. Case reports

We included studies that analysed the same individuals if they analysed different exposures or outcomes. Where more than one study analysed the same individuals using the same outcomes and exposures, only one study was included based on the study with the greatest focus and amount of information on the relationship between SES and GI infections.

Three search strategies were used to identify relevant literature. Electronic searching of three databases was performed: MEDLINE (Ovid); Scopus and Web of Science Core Collection. Search terms were piloted prior to selection and comprised specific GI infections and symptom-based terms, socioeconomic and inequality terms and developed countries of interest (Table A in S1 File). The results were restricted to publications that used data collected after 1980, to ensure the results were as relevant as possible to the present day.

Secondly, we searched the reference lists of studies selected for inclusion in the review to identify relevant articles that were not captured via electronic searching. Finally, grey literature was searched by entering the terms “gastrointestinal infection”, “gastroenteritis”, “diarrhoea”, “diarrhea”, “socioeconomic”, “social class”, “income”, and “deprivation” into the Google internet search engine and Google Scholar search application. The first 100 results from each search were screened for inclusion.

Titles and abstracts of the publications were screened independently by two authors (NA and TR) to ensure consistency in the application of inclusion/exclusion criteria. Discrepancies were discussed and resolved through a consensus process. The full text for studies deemed relevant after title and abstract screening, were sought and screened in the same way.

### Data analysis

Data were extracted into a standardised Excel spreadsheet by one reviewer and were checked by the second reviewer. Data extracted included: aim/hypothesis; study design; level of analysis; country; sample size; age; measurement of GI infection; measurement of SES; covariates and results. For studies where quantitative data were reported in text form only, authors were contacted to obtain the relevant data.

Risk of bias and quality assessment of the studies were conducted by the review team independently and then reconciled. The Liverpool University Quality Assessment Tool (LQAT) was used for this review, which allowed for the methodological quality of the studies to be assessed using a tool specific to each study design.[24] LQAT incorporates a star rating system to assess and quantify absence of bias, misclassification and confounding. It has been used in a number of other reviews[25,26] and has been independently evaluated against other quality assessment tools.[27] Discrepancies between reviewers in the quality assessment of the studies were discussed, re-examined and resolved.

Both harvest plots and meta-analysis were used to synthesise the data. Harvest plots were created to display and summarise the results of the studies and the subgrouping graphically.[28] Each reported association between SES and GI infection was represented by a single bar. The height of the bars were used to indicate the quality score of the studies from which the associations arose, so that the strength of the evidence could be determined, and greater weight given to conclusions drawn from the most methodologically robust and reliable studies. An inclusive strategy was used for the harvest plots, allowing all studies to be captured graphically, irrespective of whether quantitative estimates were provided. The findings from the harvest plots were used to inform the methods used in the meta-analysis and lead to potential explanations for the contrasting findings observed in the literature.

Subgroup analyses were performed on study design factors and potential moderating factors of the relationship identified a priori,[23] including: pathogen type (based on predominant mode of transmission–foodborne (*Campylobacter*, *Salmonella*, *Yersinia enterocolitica*); person-to-person (viral GI infections, *Shigella*); waterborne (*Giardia*, *Cryptosporidium*); environmental (Shiga toxin-producing *Escherichia coli* [STEC]); age; country (based on climate and level of development); methods used to sample GI infection cases; methods used to measure SES; and level of analysis (aggregate or individual). Predominant mode of transmission for each pathogen was assigned following consultation with experts in GI pathogens and based on available literature, however it is noted that for some pathogens, most notably STEC, there are multiple transmission routes. The Human Development Index[29] was used to classify the countries by relative level of development, and climate zones were assigned based on the Köppen system.[30] Separate tables and harvest plots were created for each subgroup, detailing the number of studies finding a positive, negative and no association, across the categories of the subgroup.

Meta-analyses were conducted in R (version 3.3.1) using an inverse variance random-effects model on combined results. Where necessary, standard methods were used to calculate the risk ratios and confidence intervals.[31] Where studies analysed the same cases, or provided numerous estimates for the relationship between SES and GI infection, only one estimate was retained in the meta-analysis to avoid the double counting of cases. For example, where studies provided estimates for numerous SES measures, the most commonly used SES measure across all of the studies (education level) was chosen if available. Estimates that were adjusted for potential confounding variables, such as age and sex, were chosen over univariate estimates. Eleven studies provided more than one estimate but the cases used for each estimate were considered independent of each other, so all estimates were included in the meta-analysis. Studies that belonged to this category included those with estimates for children and adults, and estimates for different pathogens since it was assumed that it would be unlikely for a case to be infected with more than one pathogen. However, a potential issue when including multiple estimates from single studies in random-effects meta-analyses, is the within-study variability of the different estimates would be treated as between-study variability, therefore studies with multiple estimates would have received a disproportionately high weight in the pooled estimate. Therefore fixed-effect meta-analyses were used to combine estimates from the same study, allowing these pooled estimates to be combined with the remaining studies using random-effects meta-analysis.[31]

Statistical heterogeneity was assessed by applying the I^2^ statistic with values of 30 to 60%, 50 to 90% and 75 to 100% used to denote moderate, substantial and considerable levels of heterogeneity, respectively.[31] Random-effects meta-regression[32,33] and subgroup meta-analyses were conducted to investigate potential moderating factors of the relationship between SES and GI infections, guided by the harvest plot findings. Sensitivity analysis on the basis of study quality was conducted to explore the robustness of the meta-analysis. Small study effects, which can be viewed as an indication of publication bias, were assessed using a funnel plot.

## Results

**Fig 1:**
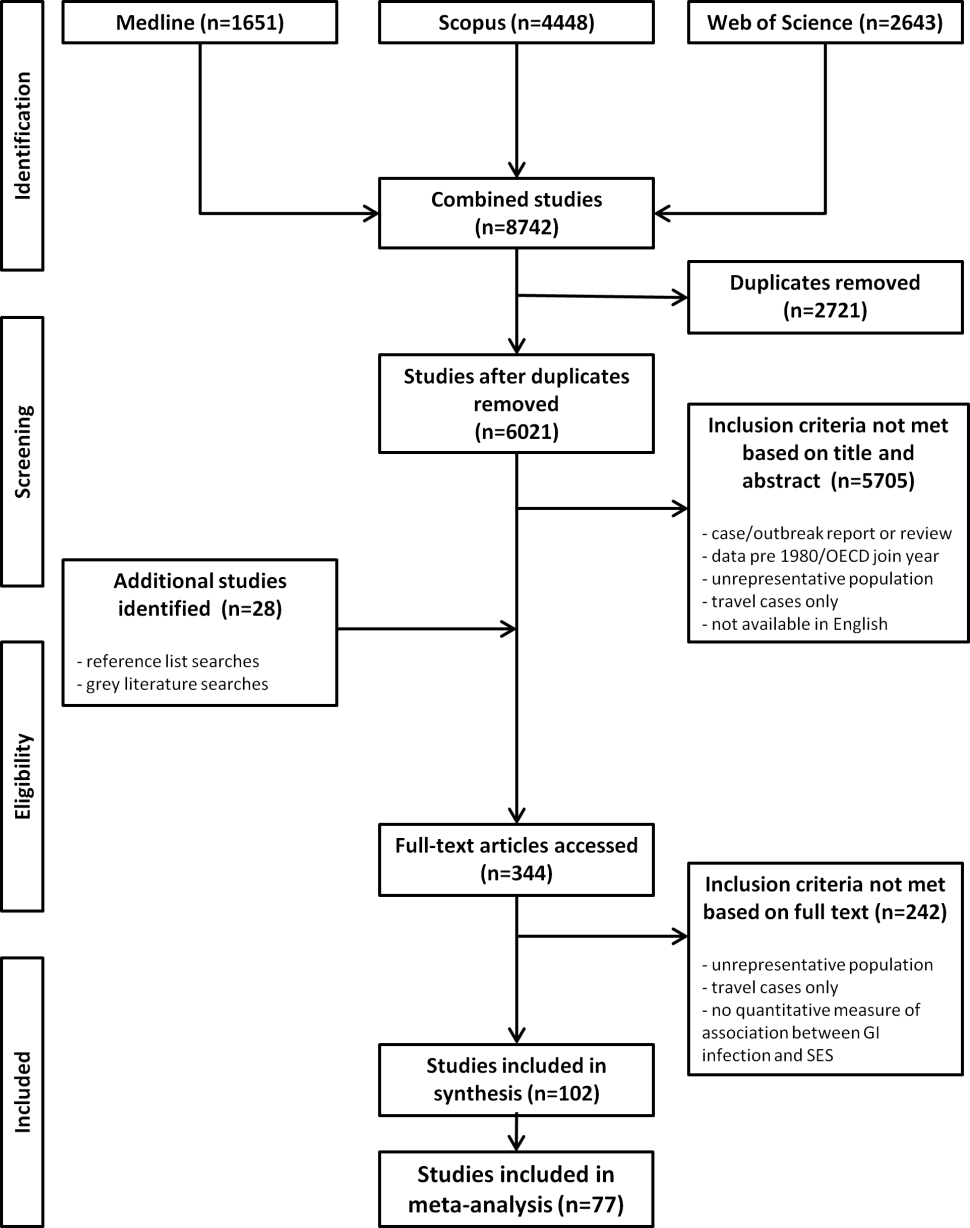
Flow diagram of studies included in the systematic review and meta-analysis.

Following duplicate removal, the database search identified 6021 citations, and 344 were full-text screened. Of these, 102 were regarded as eligible for inclusion in the review and 77 were eligible for inclusion in the meta-analysis (Fig 1). Table 2 shows the summary characteristics of the included studies. The majority of studies were conducted in Europe, had ecological study designs, used laboratory records to identify GI infection cases, and did not stratify by age. Education level was identified as the most commonly used measure of SES across the studies. Full details of the studies can be found in Table B in S1 File.

**Table 2:** Characteristics of included studies.

Study characteristics	Studies* (number)
**Total**	102
**Year of publication**	
Before 2000	17
2000–2005	15
2006–2010	38
After 2010	32
**Level of analysis**	
Individual	59
Area	43
**Region**	
Asia	3
Europe	49
North America	34
Oceania	16
**Sample size**	
<200	3
200–1000	25
1001–5000	15
5001–10000	9
10001–100000	5
>100000	45
**Age category**	
Children (<18 years old)	27
Adults	8
Mixed	61
Not stated	6
**Gastrointestinal infection outcome**	
Acute GI infection (syndromic)	41
Campylobacteriosis	20
Cryptosporidiosis	4
Giardiasis	3
Hepatitis A	3
Listeriosis	1
Norovirus	1
Rotavirus	3
Salmonellosis	8
Shigellosis	3
Shiga toxin-producing *E*. *coli* infection	4
*Yersinia enterocolitica*	1
Multiple pathogens	10
**Gastrointestinal infection measure**	
Population-based survey	30
General practice (GP) presentation	5
Hospital admission	13
Laboratory records	52
Multiple measures	2
**Socioeconomic status measure**	
Deprivation	17
Education	22
Employment	7
Income	10
Occupation	8
Social class	10
Multiple measures	28
**Study design**	
Case-control	25
Cohort	16
Cross-sectional	18
Ecological	43
**Quality**	
High	19
Medium	27
Low	56

*See S1 File for full overview of included studies.

The majority of the studies were graded as low quality (n = 56). Of these there were four cross-sectional, 35 ecological, eight cohort and nine case-control studies. Twenty-seven studies were graded as being of medium quality, including seven cross-sectional, four ecological, four cohort and 12 case-control studies. Finally, 19 studies were graded as high quality, seven cross-sectional, four ecological, four cohort and four case-control studies.

**Fig 2:**
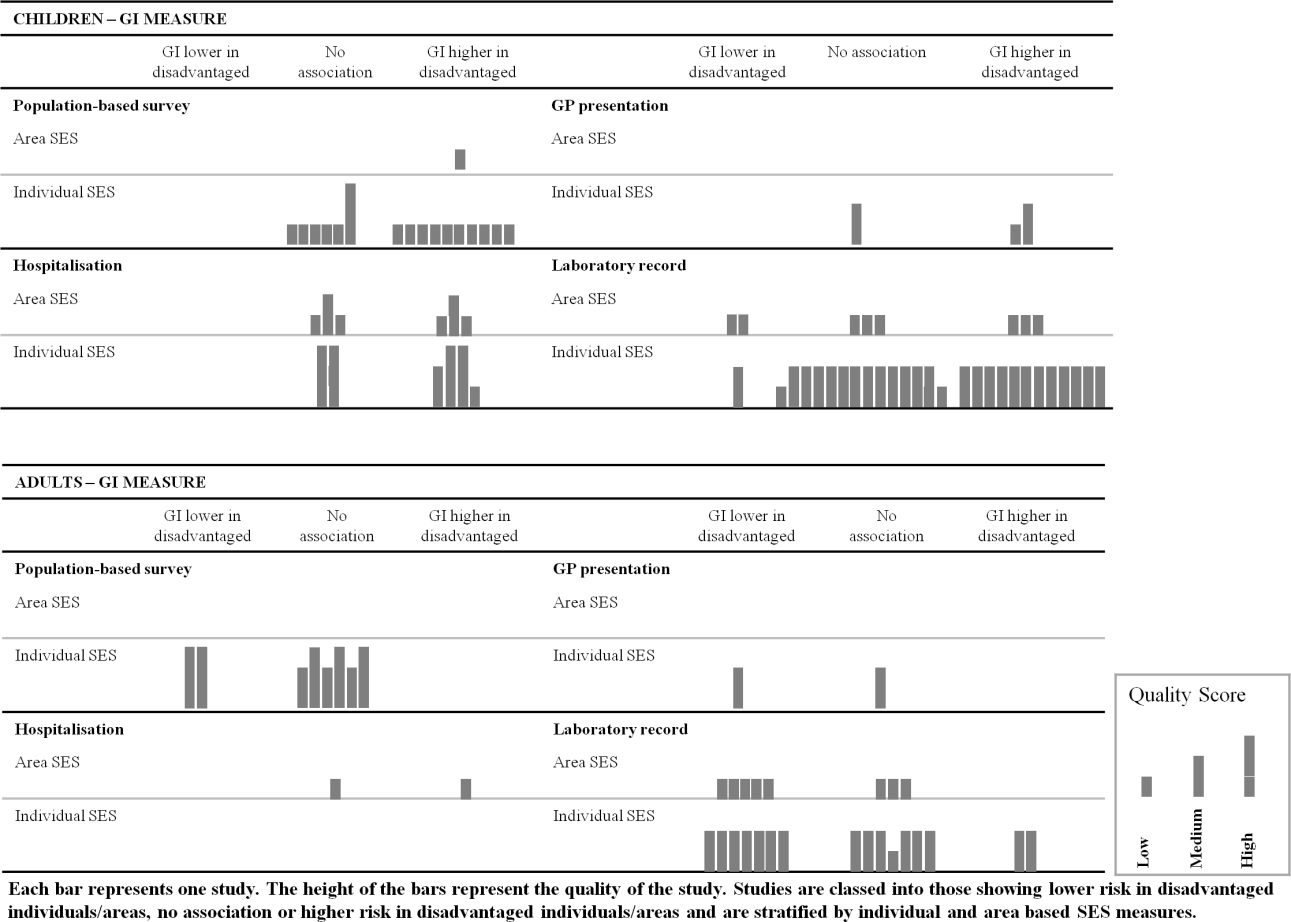
Harvest plot for risk of GI infection by SES, stratified by age, GI infection measure and SES measure.

Fig 2 shows the harvest plot for GI infection by SES, stratified by age, method of identifying GI infection cases, and SES measure. Similarly, a harvest plot stratified by age, pathogen transmission route and SES measure is presented in Fig A in S1 File. Of the 102 studies included, there were 103 point estimates for the association between SES and GI infection risk for adults or children specifically, and these point estimates were represented graphically as bars in the harvest plot (Fig 2). In the harvest plots, each bar represents one study. The height of the bar represents the quality of the study. Studies are classed into those showing lower risk in disadvantaged individuals/areas, no association or higher risk in disadvantaged individuals/areas.

The harvest plot (Fig 2) illustrates that the relationship between SES and GI infection varied with age.

The results for children by method of GI data collection are presented in the upper half of Fig 2. There was a clear social patterning for children in the reviewed studies, showing higher risk of GI infection in disadvantaged children or no association between GI infection and SES; although most studies were of low quality. With the exception of a small number of laboratory record studies, none of the studies found a lower risk of GI infection in disadvantaged children. The Harvest Plot in Fig 2 also shows that there were gaps in the literature using GP presentation to explore the relationship between GI infection and SES.

The results for adults by method of GI data collection are presented in the lower half of Fig 2. The pattern for adults different from that for children, with most studies weighted towards lower risk of GI infection in disadvantaged adults or no association. There were far fewer studies exploring the association between GI infection and SES in adults and notable gaps in studies exploring the association using hospitalisation or GP presentation data and only low quality studies using hospitalisation data.

Amongst the point estimates based on cases with a laboratory report, pathogens were grouped into predominant mode of transmission (displayed in Figure A in S1 File). There was no clear modifying role of pathogen type (based on the predominant route of transmission) on the relationship between SES and GI infection risk, although there were some differences by age. For children (upper half of Figure A in S1 File), as for the previous harvest plot, the results were socially patterned towards higher risk of foodborne (*Campylobacter*, *Salmonella*, *Yersinia enterocolitica*) and person-to-person (viral GI infections, *Shigella*) GI infection in disadvantaged children and no association for waterborne infections (*Giardia*, *Cryptosporidium*). Only three studies explored the relationship between predominantly environmental GI infections (STEC) and SES and none were of high quality. For adults (lower half of Figure A in S1 File), there were also notable gaps in studies exploring the relationship in environmental or waterborne GI infections. There was a clear pattern with studies reporting lower risk in more disadvantaged adults or no association for studies exploring the relationship between predominantly foodborne GI infections and SES, and these studies were generally of medium quality.

No clear difference was observed in the relationship between SES and GI infection, when comparing point estimates based on area and individual SES measures, or when comparing point estimates from different countries (based on level of development or climate) (data not shown).

Of the 102 studies included in this systematic review, 77 studies were included in the meta-analysis. These 77 studies contributed 83 effect estimates. Of the 25 studies that could not be included in a meta-analysis, 15 did not provide sufficient quantitative data, six did not use a dichotomous outcome and four analysed the same cases as other studies (Table B in S1 File). Since age was highlighted as a key potential effect modifier in the harvest plots, estimates from the same study stratified by age were retained individually in the meta-analysis to allow for the investigation of this variable.

The pooled risk ratio for GI infection comparing low verses high SES for all studies combined was 1.06 (95% CI 0.95–1.19), with considerable statistical heterogeneity (I^2^ 99.08%). Potential effect modifiers and sources of heterogeneity were further explored in a multivariate random-effects meta-regression in an attempt to quantitatively explain some of the heterogeneity. In univariate meta-regression, the risk of GI infection for low compared to high SES was on average significantly higher among studies that analysed hospitalised cases, and non-significantly higher among studies that analysed cases identified via population-based surveys and general practices, compared to studies that analysed laboratory recorded cases (Table 3). Amongst studies using laboratory records, the risk of GI infection for low compared to high SES was significantly lower among studies that analysed environmental pathogens, and significantly higher among studies that analysed person-to-person pathogens, compared to studies that analysed foodborne pathogens. The risk of GI infection between low and high SES groups was not statistically significantly different between studies conducted in countries with different climates and levels of development. Additionally, the risk of GI infection for low versus high SES was non-significantly lower among studies that used area-level compared to individual-level SES measures.

**Table 3:** Univariate and multivariate random-effects meta-regression for GI infection risk between low and high SES groups.

		Univariate RR (95% CI)	Multivariate RR (95% CI)	Number observations
**Method of sampling GI infection cases**	Laboratory records	1 (ref)	1 (ref)	43
	Population-based survey	1.11 (0.85–1.44)	1.04 (0.75–1.43)	23
	GP presentation	1.18 (0.71–1.94)	1.02 (0.62–1.69)	5
	Hospital admissions	1.49 (1.08–2.07)*	1.24 (0.88–1.73)	12
**SES measure**	Individual level	1 (ref)	1 (ref)	50
	Area level	0.87 (0.69–1.09)	0.92 (0.70–1.22)	33
**Age of participants**	Adult	1 (ref)	1 (ref)	14
	Mixed ages	1.17 (0.88–1.54)	1.22 (0.90–1.66)	42
	Child	1.89 (1.40–2.55)***	1.87 (1.35–2.59)***	27
**Country Human Development Index**^a^	Upper tertile	1 (ref)	1 (ref)	39
	Middle tertile	0.98 (0.76–1.25)	1.09 (0.84–1.41)	30
	Lower tertile	1.04 (0.73–1.49)	0.88 (0.62–1.25)	14
**Country climate**	Temperate/Mediterranean	1 (ref)	1 (ref)	62
	Arid	1.05 (0.69–1.61)	1.01 (0.67–1.52)	7
	Snow	0.81 (0.60–1.10)	0.89 (0.67–1.19)	14
**Pathogen** **type**^b^	Foodborne	1 (ref)	-	28
	Waterborne	0.73 (0.46–1.14)	-	8
	Environmental	0.46 (0.23–0.91)*	-	3
	Person-to-person	1.65 (1.05–2.59)*	-	7

CI = confidence interval; GI = gastrointestinal; ref = reference category; RR = ratio of risk ratios; SES = socioeconomic status

a Higher values indicate higher level of human development.

b Not all studies analysed specific pathogens, therefore this variable was not entered into the multivariate model.

*p <0.05.

**p <0.01.

***p <0.001.

In multivariate meta-regression (excluding pathogen type since not all studies analysed specific pathogens), age was identified as the only statistically significant potential effect modifier; the risk ratios for GI infection between low and high SES groups observed by studies that analysed children, were on average 1.87 (95% CI 1.35–2.59) times the risk ratios observed by studies that focused on adults (Table 3).

**Fig 3:**
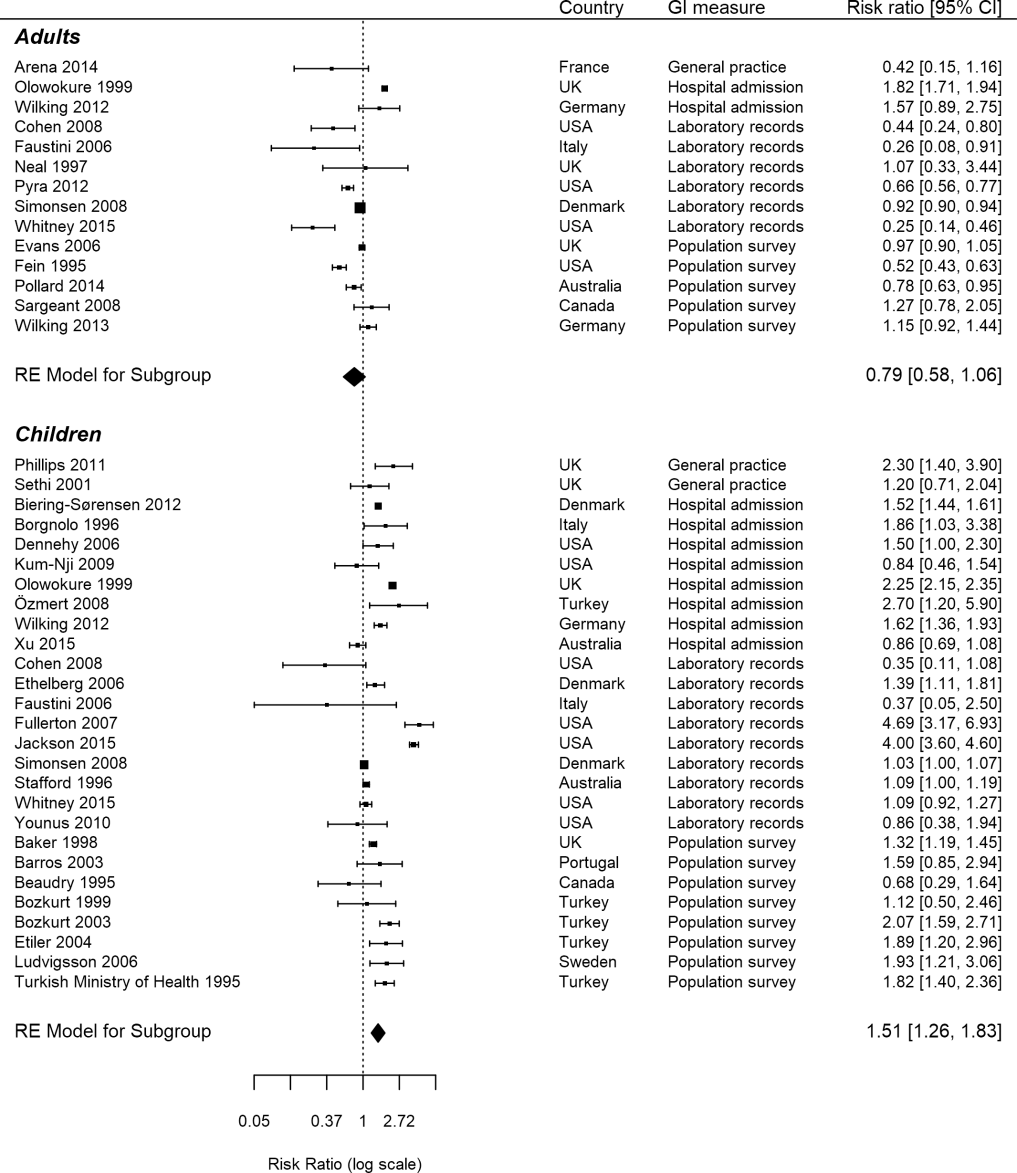
Forest plot for studies stratified by age.

A forest plot for the studies stratified by age is shown in Fig 3. For children, the pooled risk ratio was 1.51 (95% CI 1.26–1.83) with I^2^ 97.87%. For adults, the pooled risk ratio was 0.79 (95% CI 0.58–1.06) with I^2^ 98.64%. In sensitivity analyses, the results were similar when restricting to studies of high and medium quality only.

Two main outliers were identified in the forest plot (Fig 3; Jackson et al.[34], Fullerton et al.[35]). Both of these studies were conducted in the United States using laboratory records.

A contour-enhanced funnel plot[36,37] was produced to assess publication bias (Figure B in S1 File). Points within the plot appeared largely symmetrical, indicating that publication bias was unlikely.

## Discussion

In this systematic review and meta-analysis of observational studies from developed countries we found evidence of an association between lower SES and a higher risk of GI infections for children, but no association in adults. Overall, age explained a small proportion of the heterogeneity observed across the studies as a whole.

To the best of our knowledge, this study provides the first meta-analysis on this topic. We included a broad range of study designs and data sources, as well as definitions of GI infections. We used harvest plots[28] to summarise all studies, not exclusively studies with a quantitative estimate. This allowed the exploration of heterogeneity and provided important insights to inform our meta-analysis. Selection bias was mitigated by double screening throughout.

We explored the potential for publication bias, and this was not evident in our funnel plot. Subgroup analyses were defined a priori[23] which minimised the potential issues of performing multiple analyses of the data. Furthermore, these results reflect trends in inequalities of GI infections across numerous developed countries, adding confidence that the results may be generalisable.

Our review included syndromic definitions of GI infections in the absence of laboratory confirmation. We were hence able to identify literature on the burden of symptoms by SES and capture population groups who may not seek healthcare for their illness and consequently may not be included in studies which use laboratory data to identify cases. This was particularly important for this review as the decision to seek healthcare may be related to SES.

To explore sources of heterogeneity, stratified meta-analyses and meta-regression were performed on the basis of factors mentioned in the literature. Despite this, a large amount of heterogeneity remains unexplained. As seen in the forest plot, effect estimates were similar; however there were several outliers with wide confidence intervals combined with studies with narrow confidence intervals, which may provide some explanation for the extreme statistical heterogeneity observed. It is possible that factors that could not be adjusted for may explain the high residual heterogeneity. The studies covered a broad range of healthcare systems, with individual biases and caveats. SES was measured in numerous ways, and categorisation of low and high SES may have differed considerably between studies. The primary aims of the individual studies varied, as did the variables used to statistically adjust the associations between SES and GI infection risk. Further, the studies were conducted in socioeconomically diverse countries, including countries that have been in transition between developing and developed e.g. Turkey, which could potentially limit the interpretation of the results. However, our analysis of level of country development as a source of heterogeneity showed no statistically significant difference between studies conducted in countries with different levels of development. It should be noted, however, that the large amount of heterogeneity may have reduced the power to detect statistically significant modifiers in the meta-regression (Table 3), and therefore non-significance should not necessarily be interpreted as evidence that a potential modifier had no effect on the relationship between SES and risk of GI infection.[38]

Non-English language studies were excluded due to time limitations and costs of translating studies, and it is possible that bias may have been introduced by grouping the pathogen-specific studies by predominant mode of transmission, particularly for pathogens such as STEC which have multiple modes of transmission; however we consulted experts in GI pathogens for advice on the most appropriate groupings, and sensitivity analysis showed similar results when reclassifying STEC as a predominantly foodborne pathogen (data not shown). There were many ecological studies, studies conducted in Europe and studies assessed as generally low quality. Additionally, there were a few studies that focused on individual pathogens and stratified analyses by age. A number of studies had potential for bias due to study design; such as case-control studies, several of which selected controls based on the geographical residence of cases or through case-nomination, thereby potentially biasing the relationship between SES and GI infections towards the null. However, the results were similar when sensitivity analyses were conducted excluding studies which controlled for or matched by SES.

Despite the remaining heterogeneity, the evidence in this systematic review suggests that the relationship between SES and GI illness varies with age, with disadvantaged children at greater risk of GI infection compared to more advantaged children. There are no other systematic reviews that have addressed this topic in developed countries. Newman et al.[22] undertook a systematic review of the association between SES and foodborne illness, a subset of GI infections; however they did not look at differences by age or different levels of healthcare reporting such as hospitalisation. Our results are in line with those of Newman et al.[22] for foodborne and laboratory confirmed pathogen-specific results, in that there were no consistent trends across all studies or pathogens for a single SES measure, perhaps indicating weakness in the measures of social class or differential effects of SES by pathogen type.[39]

We can speculate that children may be more likely to be taken to seek medical help regardless of SES, so the higher risk of GI infection seen in children might reflect real differences by SES, rather than bias due to differential healthcare seeking behaviour. Our differing findings for children compared with adults could also reflect differential exposures or immunity by SES in children. Within developing countries, C*ampylobacter* is almost exclusively seen in disadvantaged children[40–43] while adults are rarely infected or identified,[44] potentially due to different healthcare seeking behaviour. This pattern is also seen for other bacteria and parasites.[45,46] We hypothesise that disadvantaged children are more exposed to these GI infections in childhood but that re-exposure leads to better immunity and subsequent asymptomatic infection later in life compared with their more advantaged counterparts.

Of note, the majority of studies that analysed hospital admission cases also analysed children only, making it difficult to separate out the potential modifying effects of these variables. In univariate meta-regression the risk of GI infection for low compared to high SES was significantly higher among studies that analysed hospitalised cases, compared to studies that analysed laboratory recorded cases, however this association was attenuated and rendered non-significant following adjustment for age in multivariate analysis (Table 3). Risk of person-to-person GI infection for low compared to high SES was significantly higher compared to the risk of foodborne GI infection in univariate meta-regression and risk of environmental GI infection was significantly lower. We recommend that future research investigating the association between SES and GI infection risk, should provide results stratified by adult and child age groups wherever possible, and additional insight may be gleaned by investigating the potential modifying role of pathogen type on the association.

To conclude, this systematic review finds that, in developed countries, disadvantaged children, but not adults, are at greater risk of GI infection compared to their more advantaged counterparts. Strategies to improve childhood socioeconomic conditions are likely to reduce the burden of GI illness. Additional high quality research is needed to investigate the association between SES and GI infection risk which stratifies results by age and pathogen type. Gaining greater insight into this relationship will help to inform policies to reduce the health inequalities identified.

## Supporting information

S1 File Supporting information File 1. (PDF)

S2 File PRISMA checklist. (PDF)
