# Supplementary material for: Relationship between socioeconomic status and gastrointestinal infections in developed countries: A systematic review and meta-analysis
Source: PLoS One. 2018 Jan 23;13(1):e0191633. doi: 10.1371/journal.pone.0191633 (PMC5779704; doi:10.1371/journal.pone.0191633)
Supplement: S1 File — (PDF) [file pone.0191633.s001.pdf]

Supplementary Information File

Table A: Search terms

Medline (Ovid)

#1. Exp Socioeconomic Factors/
#2. Education*.mp.
#3. Exp Employment/
#4. Income*.mp.
#5. Occupation*.mp.
#6. Poverty.mp.
#7. Poorest.mp.
#8. exp Social Class/
#9. Inequalit*.mp.
#10. Socioeconomic*.mp.
#11. Depriv*.mp.
#12. Disadvantag*.mp.
#13. Salary.mp.
#14. Underprivileged.mp.
#15. Social determinant*.mp.
#16. (Social adj1 factor*).mp
#17. Socio*.mp

#18. 1 or 2 or 3 or 4 or 5 or 6 or 7 or 8 or 9 or 10 or 11 or 12 or 13 or 14 or 15 or 16 or 17

#19. exp Norovirus/
#20. Acute gastroenteritis.mp.
#21. infectious intestinal disease*.mp.
#22. gastrointestinal infection*.mp.
#23. exp Diarrhea/
#24. Rotavirus.mp.
#25. gastrointestinal pathogen*.mp.
#26. gastrointestinal bacteria.mp.
#27. enteric infection*.mp.
#28. diarrh*.mp.
#29. stomach flu.mp.
#30. gastric flu.mp.
#31. stomach bug*.mp.
#32. stomach virus*.mp.
#33. Exp Campylobacter/
#34. Exp Escherichia coli/
#35. Enterobacteriaceae Infection*.mp.
#36. Dysentery, Bacillary.mp
#37. Exp Escherichia coli Infections/
#38. Yersinia enterocolitica.mp.
#39. Exp Salmonella Infections/
#40. Exp Cryptosporidiidae/
#41. Exp Salmonella/
#42. Exp Shigella/
#43. Exp Giardia/
#44. Escherichia coli.mp.
#45. Exp Listeria/

#46. Small round structured virus*.mp.
#47. Winter vomiting disease*.mp.
#48. Sapovirus.mp.
#49. Caliciviridae.mp.
#50. VTEC.mp.
#51. STEC.mp.
#52. exp Foodborne Diseases/
#53. Food poisoning*.mp.
#54. Scombro*.mp.
#55. Clostridium perfringens.mp.
#56. Bacillus cereus.mp.
#57. Hepatitis A.mp.
#58. Hepatitis E.mp.

#59. 19 or 20 or 21 or 22 or 23 or 24 or 25 or 26 or 27 or 28 or 29 or 30 or 31 or 32 or 33 or 34 or 35 or 36 or 37 or 38 or 39 or 40 or 41 or 42 or 43 or 44 or 45 or 46 or 47 or 48 or 49 or 50 or 51 or 52 or 53 or 54 or 55 or 56 or 57 or 58

#60. exp Australia/
#61. exp Austria/
#62. exp Belgium/
#63. exp Canada/
#64. exp Chile/
#65. exp Czech Republic/
#66. exp Denmark/
#67. exp Estonia/
#68. exp Finland/
#69. exp France/
#70. exp Germany/
#71. exp Greece/
#72. exp Hungary/
#73. exp Iceland/
#74. exp Ireland/
#75. exp Israel/
#76. exp Italy/
#77. exp Japan/
#78. exp Korea/
#79. exp Luxembourg/
#80. exp Mexico/
#81. exp Netherlands/
#82. exp New Zealand/
#83. exp Norway/
#84. exp Poland/
#85. exp Portugal/
#86. exp Slovak Republic/
#87. exp Slovenia/
#88. exp Spain/
#89. exp Sweden/
#90. exp Switzerland/
#91. exp Turkey/
#92. exp United Kingdom/
#93. exp United States/

#94. 60 or 61 or 62 or 63 or 64 or 65 or 66 or 67 or 68 or 69 or 70 or 71 or 72 or 73 or 74 or 75 or 76 or 77 or 78 or 79 or 80 or 81 or 82 or 83 or 84 or 85 or 86 or 87 or 88 or 89 or 90 or 91 or 92 or 93

#95. 18 and 59 and 94

	Scopus - TITLE-ABS-KEY	WOS Core Collection - Topic
#1.	"Career mobility"	
#2.	Poverty	
#3.	"Social class*"	
#4.	"Social mobility"	
#5.	Education*	
#6.	Employment	
#7.	Unemployment	
#8.	Income*	
#9.	Occupation*	
#10.	Poor*	
#11.	Inequalit*	
#12.	Depriv*	
#13.	Disadvantag*	
#14.	Salary	
#15.	Underprivileged	
#16.	"Social determinant*"	
#17.	Social pre/1 factor*	Social near/1 factor*
#18.	Socio*	
#19.	1 or 2 or 3 or 4 or 5 or 6 or 7 or 8 or 9 or 10 or 11 or 12 or 13 or 14 or 15 or 16 or 17 or 18	
#20.	Norovirus	
#21.	"Norwalk virus"	
#22.	"Acute gastroenteritis"	
#23.	"Infectious intestinal disease*"	
#24.	"Gastrointestinal infection*"	
#25.	Rotavirus	
#26.	"Gastrointestinal pathogen*"	
#27.	"Gastrointestinal bacteria"	
#28.	"Enteric infection*"	
#29.	Diarrh*	
#30.	"Stomach flu"	
#31.	"Gastric flu"	
#32.	"Stomach bug*"	
#33.	"Stomach virus*"	
#34.	"Escherichia coli"	
#35.	"Enterobacteriaceae Infection*"	
#36.	Dysentery Bacillary	
#37.	"Yersinia enterocolitica"	

#38. “paratyphoid fever”
#39. “typhoid fever”
#40. “Small round structured virus*”
#41. “Winter vomiting disease*”
#42. Sapovirus
#43. Caliciviridae
#44. Campylobacter*
#45. Cryptospor*
#46. Salmonell*
#47. Shigell*
#48. Giardia*
#49. Listeri*
#50. VTEC
#51. STEC
#52. “Foodborne Disease*”
#53. Botulism
#54. “Staphylococcal Food Poisoning*”
#55. “Food poisoning*”
#56. Scombro*
#57. “Clostridium perfringens”
#58. “Bacillus cereus”
#59. “Hepatitis A”
#60. “Hepatitis E”

#61. 20 or 21 or 22 or 23 or 24 or 25 or 26 or 27 or 28 or 29 or 30 or 31 or 32 or 33 or 34 or 35 or 36 or 37 or 38 or 39 or 40 or 41 or 42 or 43 or 44 or 45 or 46 or 47 or 48 or 49 or 50 or 51 or 52 or 53 or 54 or 55 or 56 or 57 or 58 or 59 or 60

#62. Australia*
#63. “New South Wales”
#64. “Northern Territory”
#65. Queensland
#66. Tasmania
#67. Victoria
#68. Austria
#69. Belgium
#70. Canada
#71. Alberta
#72. “British Columbia”
#73. Manitoba
#74. “New Brunswick”
#75. “Newfoundland and Labrador”
#76. “Northwest Territories”
#77. “Nova Scotia”
#78. Nunavut
#79. Ontario
#80. “Prince Edward Island”
#81. Quebec
#82. Saskatchewan

#83. “Yukon Territory”
#84. Chile
#85. “Czech Republic”
#86. Denmark
#87. Greenland
#88. Estonia
#89. Finland
#90. France
#91. Paris
#92. Germany
#93. Berlin
#94. Greece
#95. Hungary
#96. Iceland
#97. Ireland
#98. Israel
#99. Italy
#100. Rome
#101. Sicily
#102. Japan
#103. Tokyo
#104. Korea
#105. Seoul
#106. Luxembourg
#107. Mexico
#108. Netherlands
#109. “New Zealand”
#110. Norway
#111. Svalbard
#112. Poland
#113. Portugal
#114. “Slovak Republic”
#115. Slovakia
#116. Slovenia
#117. Spain
#118. Sweden
#119. Switzerland
#120. Turkey
#121. “United Kingdom”
#122. “Great Britain”
#123. “Channel Islands”
#124. Guernsey
#125. England
#126. London
#127. Scotland
#128. Hebrides
#129. Wales
#130. “United States”
#131. “Appalachian Region”

#132. Alabama
#133. Georgia
#134. Kentucky
#135. Maryland
#136. “New York”
#137. Carolina
#138. Ohio
#139. Pennsylvania
#140. Tennessee
#141. Virginia
#142. “Great Lakes Region”
#143. Illinois
#144. Chicago
#145. Indiana
#146. Michigan
#147. Minnesota
#148. Wisconsin
#149. “Mid-Atlantic Region”
#150. Delaware
#151. “District of Columbia”
#152. Baltimore
#153. “New Jersey”
#154. Philadelphia
#155. Iowa
#156. Kansas
#157. Missouri
#158. Nebraska
#159. Dakota
#160. Oklahoma
#161. “New England”
#162. Connecticut
#163. Maine
#164. Massachusetts
#165. Boston
#166. “New Hampshire”
#167. “Rhode Island”
#168. Vermont
#169. Idaho
#170. Montana
#171. Oregon
#172. Washington
#173. Wyoming
#174. “Pacific States”
#175. Alaska
#176. California
#177. “Los Angeles”
#178. “San Francisco”
#179. Hawaii
#180. Arkansas

#181. Florida
#182. Louisiana
#183. “New Orleans”
#184. Mississippi
#185. Arizona
#186. Colorado
#187. Nevada
#188. “New Mexico”
#189. Texas
#190. Utah

#191. 62 or 63 or 64 or 65 or 66 or 67 or 68 or 69 or 70 or 71 or 72 or 73 or 74 or 75 or 76 or 77 or 78 or 79 or 80 or 81 or 82 or 83 or 84 or 85 or 86 or 87 or 88 or 89 or 90 or 91 or 92 or 93 or 94 or 95 or 96 or 97 or 98 or 99 or 100 or 101 or 102 or 103 or 104 or 105 or 106 or 107 or 108 or 109 or 110 or 111 or 112 or 113 or 114 or 115 or 116 or 117 or 118 or 119 or 120 or 121 or 122 or 123 or 124 or 125 or 126 or 127 or 128 or 129 or 130 or 131 or 132 or 133 or 134 or 135 or 136 or 137 or 138 or 139 or 140 or 141 or 142 or 143 or 144 or 145 or 146 or 147 or 148 or 149 or 150 or 151 or 152 or 153 or 154 or 155 or 156 or 157 or 158 or 159 or 160 or 161 or 162 or 163 or 164 or 165 or 166 or 167 or 168 or 169 or 170 or 171 or 172 or 173 or 174 or 175 or 176 or 177 or 178 or 179 or 180 or 181 or 182 or 183 or 184 or 185 or 186 or 187 or 188 or 189 or 190

#192. 19 and 61 and 191

Table B: Included/excluded studies

Table B1: Included studies summary

First Author	Reference Number	Year	Quality	Country/Region	Region	Age Group	Sample Size	Level – individual/ area	Study Design	Pathogen/symptom	GI Measure	SES Measure	Included in meta-analysis	Reason for exclusion from meta-analysis
Adlam	1	2011	High	New Zealand	Oceania	All	1001-5000	Individual	Cross-sectional	Acute GI infection (syndromic)	Population based survey	Income	Y	
Arena	2	2014	Medium	France	Europe	Adults	<200	Individual	Case-control	Multi-pathogen	GP Presentation	Multiple measures	Y	
Arsenault	3	2012	Low	Canada	North America	All	>100,000	Area	Ecological	Campylobacteriosis	Laboratory records	Education	Y	
Baker	4	1998	Low	UK/Ireland	Europe	Children	5001-10,000	Individual	Cohort	Acute GI infection (syndromic)	Population based survey	Education	Y	
Banatvala	5	1999	Low	UK/Ireland	Europe	All	>100,000	Area	Ecological	Salmonellosis	Laboratory records	Deprivation	Y	
Barros	6	2003	Low	Portugal	Europe	Children	200-1000	Individual	Cohort	Acute GI infection (syndromic)	Population based survey	Education	Y	
Beale	7	2010	Low	UK/Ireland	Europe	Children	5001-10,000	Area	Cohort	Acute GI infection (syndromic)	Population based survey	Social class	N	Analysed same individuals as Baker 1998
Beaudry	8	1995	Low	Canada	North America	Children	200-1000	Individual	Cohort	Acute GI infection (syndromic)	Population based survey	Social class	Y	
Bemis	9	2014	Low	United States	North America	All	>100,000	Area	Ecological	Campylobacteriosis	Laboratory records	Deprivation	Y	
Bessell	10	2010	Low	UK/Ireland	Europe	All	>100,000	Area	Ecological	Campylobacteriosis	Laboratory records	Deprivation	Y	
Biering-Sorensen	11	2012	High	Denmark	Europe	Children	>100,000	Individual	Cohort	Acute GI infection (syndromic)	Hospital admission	Education	Y	
Bless	12	2014	High	Switzerland	Europe	All	200-1000	Individual	Case-control	Campylobacteriosis	Laboratory records	Education	Y	
Borgnolo	13	1996	High	Italy	Europe	Children	200-1000	Individual	Case-control	Salmonellosis	Hospital admission	Occupation	Y	
Bozkurt	14	1999	Low	Turkey	Europe	Children	200-1000	Individual	Cross-sectional	Acute GI infection (syndromic)	Population based survey	Multiple measures	Y	
Bozkurt	15	2003	Low	Turkey	Europe	Children	200-1000	Individual	Cohort	Acute GI infection (syndromic)	Population based survey	Multiple measures	Y	
Britton	16	2010	Low	New Zealand	Oceania	Not specified	>100,000	Area	Ecological	Multi-pathogen	Laboratory records	Deprivation	Y	
Chang	17	2009	High	United States	North America	All	>100,000	Area	Ecological	Multi-pathogen	Laboratory records	Multiple measures	N	Did not use a dichotomous outcome
Cohen	18	2008	Low	United States	North	All	>100,000	Area	Ecological	Multi-pathogen	Laboratory	Income	Y	

					America						records			
Danis	[19]	2009	Low	UK/Ireland	Europe	All	200-1000	Individual	Case-control	Campylobacteriosis	Laboratory records	Employment	Y	
de Wit	[20]	2001	Medium	Netherlands	Europe	All	1001-5000	Individual	Cohort	Acute GI infection (syndromic)	Population based survey	Education	Y	
de Wit	[21]	2003	Low	Netherlands	Europe	All	200-1000	Individual	Case-control	Multi-pathogen	Population based survey	Education	N	Subset of de Wit 2001
Dennehy	[22]	2006	High	United States	North America	Children	1001-5000	Individual	Case-control	Rotavirus	Hospital admission	Education	Y	
Doorduyn	[23]	2012	High	Netherlands	Europe	All	1001-5000	Individual	Cross-sectional	Acute GI infection (syndromic)	Population based survey	Education	Y	
Doré	[24]	2004	High	Canada	North America	All	200-1000	Individual	Case-control	Salmonellosis	Laboratory records	Education	Y	
Duggirala	[25]	2005	Low	United States	North America	All	200-1000	Individual	Case-control	Hepatitis A	Laboratory records	Education	Y	
Eaton-Evans	[26]	1987	Low	Australia	Oceania	Children	<200	Individual	Cohort	Acute GI infection (syndromic)	Population based survey	Occupation	N	Insufficient quantitative data
Ethelberg	[27]	2006	Medium	Denmark	Europe	Children	1001-5000	Individual	Case-control	Acute GI infection (syndromic)	Laboratory records	Multiple measures	Y	
Etiler	[28]	2004	Low	Turkey	Europe	Children	200-1000	Individual	Cohort	Acute GI infection (syndromic)	Population based survey	Multiple measures	Y	
Evans	[29]	2006	Medium	UK/Ireland	Europe	Adults	10,001-100,000	Individual	Cross-sectional	Acute GI infection (syndromic)	Population based survey	Employment	Y	
Faustini	[30]	2006	Medium	Italy	Europe	All	<200	Individual	Case-control	Giardiasis	Laboratory records	Education	Y	
Fein	[31]	1995	High	United States	North America	Adults	1001-5000	Individual	Cross-sectional	Acute GI infection (syndromic)	Population based survey	Education	Y	
Fewtrell	[32]	1997	Low	UK/Ireland	Europe	All	>100,000	Area	Ecological	Multi-pathogen	Laboratory records	Employment	N	Did not use a dichotomous outcome
Friedman	[33]	2004	Low	United States	North America	All	1001-5000	Individual	Case-control	Campylobacteriosis	Laboratory records	Multiple measures	Y	
Fullerton	[34]	2007	Medium	United States	North America	Children	1001-5000	Individual	Case-control	Campylobacteriosis	Laboratory records	Multiple measures	Y	
Gillespie	[35]	2008	Low	UK/Ireland	Europe	All	>100,000	Area	Ecological	Campylobacteriosis	Laboratory records	Occupation	Y	
Gillespie	[36]	2010	Low	UK/Ireland	Europe	All	>100,000	Area	Ecological	Listeriosis	Laboratory records	Deprivation	Y	
Green	[37]	2006	High	Canada	North America	All	>100,000	Area	Ecological	Campylobacteriosis	Laboratory records	Social class	Y	
Gupta	[38]	2004	Low	United States	North America	All	>100,000	Area	Ecological	Shigellosis	Laboratory records	Deprivation	N	Insufficient quantitative data

Hall	[39]	2006	High	Australia	Oceania	All	5001-10,000	Individual	Cross-sectional	Acute GI infection (syndromic)	Population based survey	Multiple measures	Y	
Herikstad	[40]	2002	Medium	United States	North America	All	5001-10,000	Individual	Cross-sectional	Acute GI infection (syndromic)	Population based survey	Multiple measures	N	Analysed same individuals as Jones 2007
Hu	[41]	2009	Low	Australia	Oceania	Not specified	>100,000	Area	Ecological	Cryptosporidiosis	Laboratory records	Multiple measures	Y	
Hu	[42]	2010	Low	Australia	Oceania	Not specified	>100,000	Area	Ecological	Cryptosporidiosis	Laboratory records	Social class	N	Did not use a dichotomous outcome
Hughes	[43]	2015	Low	UK/Ireland	Europe	All	>100,000	Area	Ecological	Multi-pathogen	Laboratory records	Deprivation	Y	
Iacono	[44]	2005	High	Italy	Europe	Children	1001-5000	Individual	Cohort	Acute GI infection (syndromic)	Population based survey	Education	N	Insufficient quantitative data
Jackson	[45]	2015	Low	United States	North America	Children	>100,000	Area	Ecological	Shigellosis	Laboratory records	Deprivation	Y	
Jalava	[46]	2011	Low	Finland	Europe	All	>100,000	Area	Ecological	STEC	Laboratory records	Multiple measures	Y	
Jones	[47]	2007	Medium	United States	North America	All	10,001-100,000	Individual	Cross-sectional	Acute GI infection (syndromic)	Population based survey	Education	Y	
Kass	[48]	1992	Low	United States	North America	All	200-1000	Individual	Case-control	Salmonellosis	Laboratory records	Income	Y	
Kotloff	[49]	1988	Medium	United States	North America	Children	200-1000	Individual	Case-control	Acute GI infection (syndromic)	Hospital admission	Education	N	Insufficient quantitative data
Kum-Nji	[50]	2009	High	United States	North America	Children	200-1000	Individual	Cohort	Acute GI infection (syndromic)	Hospital admission	Employment	Y	
Kyle	[51]	2011	Low	UK/Ireland	Europe	Children	>100,000	Area	Ecological	Acute GI infection (syndromic)	Hospital admission	Deprivation	N	Did not use a dichotomous outcome
Lake	[52]	2007	Low	UK/Ireland	Europe	All	5001-10,000	Area	Ecological	Cryptosporidiosis	Laboratory records	Occupation	Y	
Lake	[53]	2009	Low	UK/Ireland	Europe	All	>100,000	Area	Ecological	Cryptosporidiosis	Laboratory records	Social class	N	Insufficient quantitative data
Lal	[54]	2012	Medium	New Zealand	Oceania	All	>100,000	Area	Ecological	Salmonellosis	Multiple measures	Deprivation	Y	
Lee	[55]	1991	Low	United States	North America	All	>100,000	Area	Ecological	Shigellosis	Laboratory records	Deprivation	N	Insufficient quantitative data
Ludvigsson	[56]	2006	Low	Sweden	Europe	Children	5001-10,000	Individual	Cohort	Acute GI infection (syndromic)	Population based survey	Education	Y	

MacRitchie	57	2013	Low	UK/Ireland	Europe	All	>100,000	Area	Ecological	Campylobacteriosis	Laboratory records	Deprivation	N	Insufficient quantitative data
Majowicz	58	2007	Medium	Canada	North America	All	5001-10,000	Individual	Cross-sectional	Acute GI infection (syndromic)	Population based survey	Multiple measures	Y	
McAteer	59	2011	High	UK/Ireland	Europe	Adults	1001-5000	Individual	Cross-sectional	Acute GI infection (syndromic)	Population based survey	Multiple measures	N	Insufficient quantitative data
McPherson	60	2009	Medium	Australia	Oceania	All	200-1000	Individual	Case-control	STEC	Laboratory records	Education	Y	
Moorin	61	2010	Medium	Australia	Oceania	All	>100,000	Area	Cohort	Acute GI infection (syndromic)	Hospital admission	Social class	Y	
Neal	62	1997	Low	UK/Ireland	Europe	Adults	200-1000	Individual	Case-control	Campylobacteriosis	Laboratory records	Social class	Y	
Nichols	63	2012	Low	UK/Ireland	Europe	All	>100,000	Area	Ecological	Campylobacteriosis	Laboratory records	Deprivation	N	Insufficient quantitative data
Odoi	64	2004	Medium	Canada	North America	Not specified	>100,000	Area	Ecological	Giardiasis	Laboratory records	Income	Y	
Olowokure	65	1999	Low	UK/Ireland	Europe	All	>100,000	Area	Ecological	Acute GI infection (syndromic)	Hospital admission	Deprivation	Y	
Özkan	66	2007	Low	Turkey	Europe	All	200-1000	Individual	Cross-sectional	Acute GI infection (syndromic)	Population based survey	Income	Y	
Özmert	67	2008	Low	Turkey	Europe	Children	200-1000	Individual	Case-control	Acute GI infection (syndromic)	Hospital admission	Education	Y	
Pardhan-Ali	68	2013	Low	Canada	North America	All	10,001-100,000	Area	Ecological	Multi-pathogen	Laboratory records	Multiple measures	N	Insufficient quantitative data
Pearl	69	2009	Low	Canada	North America	All	>100,000	Area	Ecological	STEC	Laboratory records	Income	N	Insufficient quantitative data
Penrose	70	2007	Low	United States	North America	All	>100,000	Area	Ecological	Giardiasis	Laboratory records	Income	N	Insufficient quantitative data
Phillips	71	2011	Low	UK/Ireland	Europe	Children	200-1000	Individual	case-control	Norovirus	GP Presentation	Occupation	Y	
Pockett	72	2011	Medium	UK/Ireland	Europe	Children	>100,000	Area	Ecological	Acute GI infection (syndromic)	Hospital admission	Deprivation	N	Did not use a dichotomous outcome
Pollard	73	2014	High	Australia	Oceania	Adults	1001-5000	Individual	Cross-sectional	Acute GI infection (syndromic)	Population based survey	Education	Y	
Pyra	74	2012	Low	United States	North America	Adults	>100,000	Area	Ecological	Campylobacteriosis	Laboratory records	Multiple measures	Y	
Quigley	75	2006	Medium	UK/Ireland	Europe	Children	200-1000	Individual	Case-control	Acute GI infection (syndromic)	GP Presentation	Occupation	N	Data analysed within Phillips

														2011, Rodrigues 2001 and Sethi 2001
Rind	[76]	2010	High	New Zealand	Oceania	All	>100,000	Area	Ecological	Campylobacteriosis	Laboratory records	Social class	N	Did not use a dichotomous outcome
Rodrigues	[77]	2001	Medium	UK/Ireland	Europe	All	200-1000	Individual	Case-control	Campylobacteriosis	GP Presentation	Employment	Y	
Sakuma	[78]	2006	Low	Japan	Asia	All	>100,000	Area	Ecological	STEC	Laboratory records	Income	N	Insufficient quantitative data
Sargeant	[79]	2008	Medium	Canada	North America	All	1001-5000	Individual	Cross-sectional	Acute GI infection (syndromic)	Population based survey	Multiple measures	Y	
Satterthwaite	[80]	1999	Medium	New Zealand	Oceania	All	200-1000	Individual	Case-control	Yersinia enterocolitica	Laboratory records	Education	Y	
Scallan	[81]	2004	High	UK/Ireland	Europe	All	5001-10,000	Individual	Cross-sectional	Acute GI infection (syndromic)	Population based survey	Occupation	Y	
Seo	[82]	2012	High	Korea	Asia	Not specified	>100,000	Area	Ecological	Hepatitis A	Laboratory records	Multiple measures	N	Insufficient quantitative data
Seo	[83]	2013	Medium	Korea	Asia	All	1001-5000	Individual	Case-control	Hepatitis A	Hospital admission	Multiple measures	Y	
Sethi	[84]	2001	Medium	UK/Ireland	Europe	Children	200-1000	Individual	Case-control	Rotavirus	GP Presentation	Occupation	Y	
Simonsen	[85]	2008	Medium	Denmark	Europe	All	>100,000	Individual	Cohort	Multi-pathogen	Laboratory records	Multiple measures	Y	
Spencer	[86]	2012	Low	New Zealand	Oceania	All	>100,000	Area	Ecological	Campylobacteriosis	Laboratory records	Deprivation	Y	
Stafford	[87]	1996	Low	Australia	Oceania	All	>100,000	Area	Ecological	Campylobacteriosis	Laboratory records	Social class	Y	
Stone	[88]	1994	Low	UK/Ireland	Europe	All	1001-5000	Individual	Cross-sectional	Acute GI infection (syndromic)	Population based survey	Employment	N	Insufficient quantitative data
Tam	[89]	2013	High	UK/Ireland	Europe	All	5001-10,000	Individual	Cohort	Acute GI infection (syndromic)	Population based survey	Multiple measures	Y	
Teschke	[90]	2010	Medium	Canada	North America	All	>100,000	Area	Cohort	Acute GI infection (syndromic)	Multiple measures	Income	Y	
Turkish Ministry of Health	[91]	1995	Low	Turkey	Europe	Children	1001-5000	Individual	Cross-sectional	Acute GI infection (syndromic)	Population based survey	Education	Y	
Unicomb	[92]	2008	Medium	Australia	Oceania	All	200-1000	Individual	Case-control	Campylobacteriosis	Laboratory records	Multiple measures	Y	
Van Cauteren	[93]	2012	Medium	France	Europe	All	10,001-100,000	Individual	Cross-sectional	Acute GI infection (syndromic)	Population based survey	Multiple measures	Y	

Varga	94	2013	Medium	Canada	North America	All	>100,000	Area	Ecological	Salmonellosis	Laboratory records	Multiple measures	Y	
Weisent	95	2012	Low	United States	North America	Not specified	>100,000	Area	Ecological	Campylobacteriosis	Laboratory records	Multiple measures	Y	
Whitney	96	2015	Low	United States	North America	All	>100,000	Area	Ecological	Multi-pathogen	Laboratory records	Deprivation	Y	
Wilking	97	2012	Low	Germany	Europe	All	>100,000	Area	Ecological	Rotavirus	Hospital admission	Employment	Y	
Wilking	98	2013	Medium	Germany	Europe	Adults	10,001-100,000	Individual	Cross-sectional	Acute GI infection (syndromic)	Population based survey	Income	Y	
Xu	99	2015	Low	Australia	Oceania	Children	>100,000	Area	Ecological	Acute GI infection (syndromic)	Hospital admission	Social class	Y	
Younus	100	2007	Low	United States	North America	All	>100,000	Area	Ecological	Salmonellosis	Laboratory records	Multiple measures	Y	
Younus	101	2010	Low	United States	North America	Children	200-1000	Individual	Case-control	Salmonellosis	Laboratory records	Multiple measures	Y	
Zappe Pasturel	102	2013	Low	United States	North America	All	>100,000	Area	Ecological	Campylobacteriosis	Laboratory records	Multiple measures	Y	

Table B2: Reference List for Included Studies

1. Adlam SB, Perera S, Lake RJ, Campbell DM, Williman JA, Baker MG. Acute gastrointestinal illness in New Zealand: a community study. Epidemiology and infection. 2011;139(2):302-8. doi:10.1017/S0950268810000932
2. Arena C, Amoros JP, Vaillant V, Ambert-Balay K, Chikhi-Brachet R, Jourdan-Da Silva N, et al. Acute diarrhea in adults consulting a general practitioner in France during winter: incidence, clinical characteristics, management and risk factors. BMC infectious diseases. 2014;14:574-. doi:10.1186/s12879-014-0574-4
3. Arsenault J, Berke, O., Michel, P., Ravel, A., Gosselin, P. Environmental and demographic risk factors for campylobacteriosis: Do various geographical scales tell the same story? BMC infectious diseases. 2012;12:-. doi:10.1186/1471-2334-12-318
4. Baker D, Taylor H, Henderson J. Inequality in infant morbidity: Causes and consequences in England in the 1990s. Journal of epidemiology and community health. 1998;52(7):451-8; .
5. Banatvala N, Cramp A, Jones IR, Feldman RA. Salmonellosis in North Thames (East), UK: associated risk factors. Epidemiology and infection. 1999(122):201-7.
6. Barros H, Lunet N. Association between child-care and acute diarrhea: A study in Portuguese children. Revista de saude publica. 2003;37(5):603-8; .
7. Beale N, Peart C, Kay H, Taylor G, Boyd A, Herrick D. 'ALSPAC' infant morbidity and council tax band: Doctor consultations are higher in lower bands. European journal of public health. 2010;20(4):403-8. doi:10.1093/eurpub/ckp211
8. Beaudry M, Dufour R., Marcoux S. Relation between infant feeding and infections during the first six months of life. The Journal of pediatrics. 1995;126(2):191-7; . doi:10.1016/s0022-3476(95)70544-9
9. Bemis K, Marcus R, Hadler JL. Socioeconomic status and campylobacteriosis, Connecticut, USA, 1999-2009. Emerging Infectious Diseases. 2014;20(7):1240-2. doi:http://dx.doi.org/10.3201/eid2007.131333
10. Bessell PR, Matthews L, Smith-Palmer A, Rotariu O, Strachan NJ, Forbes KJ, et al. Geographic determinants of reported human Campylobacter infections in Scotland. BMC public health. 2010;10:423. doi:10.1186/1471-2458-10-423
11. Biering-Sorensen S, Sondergaard G, Vitting Andersen K, Andersen AM, Mortensen LH. Time trends in socio-economic factors and risk of hospitalisation with infectious diseases in pre-school children 1985-2004: a Danish register-based study. Paediatric and perinatal epidemiology. 2012;26(3):226-35. doi:10.1111/j.1365-3016.2011.01255.x
12. Bless PJ, Schmutz C, Suter K, Jost M, Hattendorf J, Mausezahl-Feuz M, et al. A tradition and an epidemic: determinants of the campylobacteriosis winter peak in Switzerland. European journal of epidemiology. 2014;29(7):527-37.
13. Borgnolo G, Barbone F, Scornavacca G, Franco D, Vinci A, Iuculano F. A case-control study of Salmonella gastrointestinal infection in Italian children. Acta paediatrica. 1996;85(7):804-8.
14. Bozkurt AI, Ozgur S, Ozcirpici B. The prevalance of diseases with diarrhoea among children under five years old in different socioeconomic levels. Tr. J. of Medical Sciences. 1999;29:561-5.
15. Bozkurt AI, Ozgur S, Ozcirpici B. Association between household conditions and diarrheal diseases among children in Turkey: a cohort study. Pediatrics International. 2003;45(4):443-51.
16. Britton E, Hales S, Venugopal K, Baker MG. The impact of climate variability and change on cryptosporidiosis and giardiasis rates in New Zealand. Journal of water and health. 2010;8(3):561-71. doi:10.2166/wh.2010.049
17. Chang M, Groseclose SL, Zaidi AA, Braden CR. An ecological analysis of sociodemographic factors associated with the incidence of salmonellosis, shigellosis, and E. coli O157:H7 infections in US counties. Epidemiology & Infection. 2009;137(6):810-20. doi:http://dx.doi.org/10.1017/S0950268808001477

18. Cohen SA, Egorov AI, Jagai JS, Matyas BT, DeMaria A, Jr., Chui KK, et al. The SEEDs of two gastrointestinal diseases: socioeconomic, environmental, and demographic factors related to cryptosporidiosis and giardiasis in Massachusetts. Environmental research. 2008;108(2):185-91. doi:http://dx.doi.org/10.1016/j.envres.2008.06.009
19. Danis K, Di Renzi M, O'Neill W, Smyth B, McKeown P, Foley B, et al. Risk factors for sporadic Campylobacter infection: an all-Ireland case-control study. Euro surveillance : bulletin Europeen sur les maladies transmissibles = European communicable disease bulletin. 2009;14(7).
20. de Wit MA, Koopmans MP, Kortbeek LM, Wannet WJ, Vinje J, van Leusden F, et al. Sensor, a population-based cohort study on gastroenteritis in the Netherlands: incidence and etiology. American Journal of Epidemiology. 2001;154(7):666-74.
21. De Wit MAS, Koopmans M. P. G., Van Duynhoven Y. T. H. P. Risk Factors for Norovirus, Sapporo-like Virus, and Group A Rotavirus Gastroenteritis. Emerging Infectious Diseases. 2003;9(12):1563-70; .
22. Dennehy PH, Cortese MM, Begue RE, Jaeger JL, Roberts NE, Zhang R, et al. A case-control study to determine risk factors for hospitalization for rotavirus gastroenteritis in U.S. children. Pediatric Infectious Disease Journal. 2006;25(12):1123-31.
23. Doorduyn Y, Van Pelt W, Havelaar AH. The burden of infectious intestinal disease (IID) in the community: a survey of self-reported IID in The Netherlands. Epidemiology & Infection. 2012;140(7):1185-92. doi:http://dx.doi.org/10.1017/S0950268811001099
24. Doré K, Buxton J, Henry B, Pollari F, Middleton D, Fyfe M, et al. Risk factors for Salmonella typhimurium DT104 and non-DT104 infection: A Canadian multi-provincial case-control study. Epidemiology and infection. 2004;132(3):485-93; . doi:10.1017/s0950268803001924
25. Duggirala HJ, Hassig SE, Santana S, Rice J. Evaluation of a hepatitis A immunization program. Pediatric Infectious Disease Journal. 2005;24(11):974-8; . doi:10.1097/01.inf.0000183760.75182.01
26. Eaton-Evans J, Dugdale AE. Effects of feeding and social factors on diarrhoea and vomiting in infants. Archives of Disease in Childhood. 1987;62(5):445-8; . doi:10.1136/adc.62.5.445
27. Ethelberg S, Olesen B, Neimann J, Schiellerup P, Helms M, Jensen C, et al. Risk factors for diarrhea among children in an industrialized country. Epidemiology. 2006;17(1):24-30.
28. Etiler N, Velipasaoglu S, Aktekin M. Risk factors for overall and persistent diarrhoea in infancy in Antalya, Turkey: A cohort study. Public health. 2004;118(1):62-9; . doi:10.1016/s0033-3506(03)00132-x
29. Evans MR, Sarvotham T, Thomas DR, Howard AJ. Domestic and travel-related foodborne gastrointestinal illness in a population health survey. Epidemiology and infection. 2006;134(4):686-93; . doi:10.1017/s0950268805005790
30. Faustini A, Marinacci C, Fabrizi E, Marangi M, Recchia O, Pica R, et al. The impact of the Catholic Jubilee in 2000 on infectious diseases. A case-control study of giardiasis, Rome, Italy 2000-2001. Epidemiology and infection. 2006;134(3):649-58; . doi:10.1017/s0950268805005327
31. Fein SB, Lin CTJ, Levy AS. Foodborne illness: Perceptions, experience, and preventive behaviors in the United States. Journal of food protection. 1995;58(12):1405-11.
32. Fewtrell L, Kay D, Wyer M, O'Neill G. An investigation into the possible links between shigellosis and hepatitis A and public water supply disconnections. Public health. 1997;111(3):179-81; . doi:10.1016/s0033-3506(97)00579-9
33. Friedman CR, Hoekstra RM, Samuel M, Marcus R, Bender J, Shiferaw B, et al. Risk factors for sporadic Campylobacter infection in the United States: A case-control study in FoodNet sites. Clinical infectious diseases : an official publication of the Infectious Diseases Society of America. 2004;38 Suppl 3:S285-96. doi:10.1086/381598

34. Fullerton KE, Ingram LA, Jones TF, Anderson BJ, McCarthy PV, Hurd S, et al. Sporadic campylobacter infection in infants: a population-based surveillance case-control study. The Pediatric infectious disease journal. 2007;26(1):19-24. doi:10.1097/01.inf.0000247137.43495.34
35. Gillespie IA, O'Brien, S.J., Penman, C., Tompkins, D., Cowden, J., Humphrey, T.J. Demographic determinants for Campylobacter infection in England and Wales: Implications for future epidemiological studies. Epidemiology and infection. 2008;136(12):1717-25. doi:10.1017/S0950268808000319
36. Gillespie I, Mook P, Little C, Grant K, McLauchlin J. Human listeriosis in England, 2001-2007: association with neighbourhood deprivation. Euro Surveillance. 2010;15(27).
37. Green CG, Krause, D.O., Wylie, J.L. Spatial analysis of campylobacter infection in the Canadian province of Manitoba. International Journal of Health Geographics. 2006;5:-. doi:10.1186/1476-072X-5-2
38. Gupta A, Polyak CS, Bishop RD, Sobel J, Mintz ED. Laboratory-confirmed shigellosis in the United States, 1989-2002: epidemiologic trends and patterns. Clinical infectious diseases : an official publication of the Infectious Diseases Society of America. 2004;38(10):1372-7. doi:10.1086/386326
39. Hall GV, Kirk MD, Ashbolt R, Stafford R, Lalor K, Bell R, et al. Frequency of infectious gastrointestinal illness in Australia, 2002: Regional, seasonal and demographic variation. Epidemiology and infection. 2006;134(1):111-8. doi:10.1017/S0950268805004656
40. Herikstad H, Yang S, Van Gilder TJ, Vugia D, Hadler J, Blake P, et al. A population-based estimate of the burden of diarrhoeal illness in the United States: FoodNet, 1996-7. Epidemiology and infection. 2002;129(1):9-17.
41. Hu W, Mengersen K, Tong S. Spatial analysis of notified cryptosporidiosis infections in Brisbane, Australia. Annals of epidemiology. 2009;19(12):900-7. doi:10.1016/j.annepidem.2009.06.004
42. Hu W, Mengersen K, Tong S. Risk factor analysis and spatiotemporal CART model of cryptosporidiosis in Queensland, Australia. BMC infectious diseases. 2010;10:311. doi:10.1186/1471-2334-10-311
43. Hughes GJ, Gorton R. Inequalities in the incidence of infectious disease in the North East of England: a population-based study. Epidemiology & Infection. 2015;143(1):189-201. doi:http://dx.doi.org/10.1017/S0950268814000533
44. Iacono G, Merolla R, D'Amico D, Bonci E, Cavataio F, Di Prima L, et al. Gastrointestinal symptoms in infancy: a population-based prospective study. Dig Liver Dis. 2005;37(6):432-8. doi:10.1016/j.dld.2005.01.009
45. Jackson R, Smith D, Tabnak F, Vugia D. Disparities of shigellosis rates among California children by race/ethnicity and census tract poverty level, 2000-2010. Pediatric Infectious Disease Journal. 2015;34(8):843-7. doi:10.1097/inf.0000000000000746
46. Jalava K, Ollgren J, Eklund M, Siitonen A, Kuusi M. Agricultural, socioeconomic and environmental variables as risks for human verotoxigenic Escherichia coli (VTEC) infection in Finland. BMC infectious diseases. 2011;11:275. doi:http://dx.doi.org/10.1186/1471-2334-11-275
47. Jones TF, McMillian MB, Scallan E, Frenzen PD, Cronquist AB, Thomas S, et al. A population-based estimate of the substantial burden of diarrhoeal disease in the United States; FoodNet, 1996-2003. Epidemiology and infection. 2007;135(2):293-301. doi:10.1017/S0950268806006765
48. Kass PH, Farver TB, Beaumont JJ, Genigeorgis C, Stevens F. Disease determinants of sporadic salmonellosis in four northern California counties. A case-control study of older children and adults. Annals of epidemiology. 1992;2(5):683-96.
49. Kotloff KL, Wasserman SS, Steciak JY, Tall BD, Losonsky GA, Nair P, et al. Acute diarrhea in Baltimore children attending an outpatient clinic. Pediatric Infectious Disease Journal. 1988;7(11):753-9.

50. Kum-Nji P, Mangrem CL, Wells PJ, Herrod HG. Is environmental tobacco smoke exposure a risk factor for acute gastroenteritis in young children? Clinical Pediatrics. 2009;48(7):756-62; . doi:10.1177/0009922809332591
51. Kyle RG, Kukanova M, Campbell M, Wolfe I, Powell P, Callery P. Childhood disadvantage and emergency admission rates for common presentations in London: an exploratory analysis. Archives of Disease in Childhood. 2011;96(3):221-6. doi:http://dx.doi.org/10.1136/adc.2009.180125
52. Lake IR, Harrison FC, Chalmers RM, Bentham G, Nichols G, Hunter PR, et al. Case-control study of environmental and social factors influencing cryptosporidiosis. European journal of epidemiology. 2007;22(11):805-11.
53. Lake IR, Nichols G, Harrison FC, Bentham G, Sari Kovats R, Grundy C, et al. Using infectious intestinal disease surveillance data to explore illness aetiology; a cryptosporidiosis case study. Health & place. 2009;15(1):333-9. doi:10.1016/j.healthplace.2008.06.005
54. Lal A, Baker MG, French NP, Dufour M, Hales S. The epidemiology of human salmonellosis in New Zealand, 1997-2008. Epidemiology and infection. 2012;140(9):1685-94; . doi:10.1017/s0950268811002470
55. Lee LA, Shapiro CN, Hargrett-Bean N, Tauxe RV. Hyperendemic shigellosis in the United States: A review of surveillance data for 1967-1988. Journal of Infectious Diseases. 1991;164(5):894-900; 894.
56. Ludvigsson JF. Epidemiological study of constipation and other gastrointestinal symptoms in 8000 children. Acta Paediatrica, International Journal of Paediatrics. 2006;95(5):573-80. doi:10.1080/08035250500452621
57. MacRitchie LA, Hunter CJ, Strachan NJ. A population-based exposure assessment of risk factors associated with gastrointestinal pathogens: a Campylobacter study. Epidemiology & Infection. 2013;141(5):976-86. doi:http://dx.doi.org/10.1017/S0950268812001641
58. Majowicz SE, Horrocks J, Bocking K. Demographic determinants of acute gastrointestinal illness in Canada: a population study. BMC public health. 2007;7:8. doi:10.1186/1471-2458-7-162
59. McAteer A, Elliott, A.M., Hannaford, P.C. Ascertaining the size of the symptom iceberg in a UK-wide community-based survey. British Journal of General Practice. 2011;61(582):e1-e11. doi:10.3399/bjgp11X548910
60. McPherson M, Lalor K, Combs B, Raupach J, Stafford R, Kirk MD. Serogroup-specific risk factors for Shiga toxin-producing Escherichia coli infection in Australia. Clinical infectious diseases : an official publication of the Infectious Diseases Society of America. 2009;49(2):249-56; . doi:10.1086/599370
61. Moorin RE, Heyworth JS, Forbes GM, Riley TV. Long-term health risks for children and young adults after infective gastroenteritis. Emerging Infectious Diseases. 2010;16(9):1440-7; . doi:10.3201/eid1609.081665
62. Neal KR, Slack RCB. Diabetes mellitus, anti-secretory drugs and other risk factors for campylobacter gastro-enteritis in adults: A case-control study. Epidemiology and infection. 1997;119(3):307-11; . doi:10.1017/s0950268897008224
63. Nichols GL, Richardson, J.F., Sheppard, S.K., Lane, C., Sarran, C. Campylobacter epidemiology: A descriptive study reviewing 1 million cases in England and Wales between 1989 and 2011. BMJ open. 2012;2(4):-. doi:10.1136/bmjopen-2012-001179
64. Odoi A, Martin SW, Michel P, Holt J, Middleton D, Wilson J. Determinants of the geographical distribution of endemic giardiasis in Ontario, Canada: A spatial modelling approach. Epidemiology and infection. 2004;132(5):967-76; . doi:10.1017/s0950268804002481
65. Olowokure B, Hawker J, Weinberg J, Gill N, Sufi F. Deprivation and hospital admission for infectious intestinal diseases. Lancet. 1999;353(9155):807-8. doi:10.1016/S0140-6736(99)00611-X

66. Özkan S, Tüzün H, Görer N, Ceyhan M, Aycan S, Albayrak S, et al. Water usage habits and the incidence of diarrhea in rural Ankara, Turkey. Transactions of the Royal Society of Tropical Medicine and Hygiene. 2007;101(11):1131-5; . doi:10.1016/j.trstmh.2007.05.011
67. Özmert EN, Kilic M, Yurdakök K. Environmental tobacco smoke: Is it a risk factor for diarrhea in 6-18 months old infants? Central European journal of public health. 2008;16(2):85-6; .
68. Pardhan-Ali A, Wilson J, Edge VL, Furgal C, Reid-Smith R, Santos M, et al. Community-level risk factors for notifiable gastrointestinal illness in the Northwest Territories, Canada, 1991-2008. BMC public health. 2013;13:63. doi:http://dx.doi.org/10.1186/1471-2458-13-63
69. Pearl DL, Louie M, Chui L, Dore K, Grimsrud KM, Martin SW, et al. A multi-level approach for investigating socio-economic and agricultural risk factors associated with rates of reported cases of Escherichia coli O157 in humans in Alberta, Canada. Zoonoses & Public Health. 2009;56(8):455-64. doi:http://dx.doi.org/10.1111/j.1863-2378.2008.01193.x
70. Penrose AS, Wells EV, Aiello AE. Infectious causation of chronic disease: Examining the relationship between Giardia lamblia infection and irritable bowel syndrome. World Journal of Gastroenterology. 2007;13(34):4574-8; .
71. Phillips G, Tam CC, Rodrigues LC, Lopman B. Risk factors for symptomatic and asymptomatic norovirus infection in the community. Epidemiology & Infection. 2011;139(11):1676-86. doi:http://dx.doi.org/10.1017/S0950268810002839
72. Pockett RD, Adlard N, Carroll S, Rajoriya F. Paediatric hospital admissions for rotavirus gastroenteritis and infectious gastroenteritis of all causes in England: an analysis of correlation with deprivation. Current medical research and opinion. 2011;27(4):777-84. doi:10.1185/03007995.2011.555757
73. Pollard CM, Meng X, Williamson S, Dodds J, Binns CW. Eating out is associated with self-reported food poisoning: a Western Australia population perspective, 1998 to 2009. Public health nutrition. 2014;17(10):2270-7. doi:10.1017/s1368980013002371
74. Pyra M, Conover C, Howland J, Soyemi K. Determinants of campylobacteriosis notifications in New Zealand. Epidemiology and infection. 2012;140(11):2087-8. doi:10.1017/s0950268812000441
75. Quigley MA, Cumberland P, Cowden JM, Rodrigues LC. How protective is breast feeding against diarrhoeal disease in infants in 1990s England? A case-control study. Archives of Disease in Childhood. 2006;91(3):245-50; . doi:10.1136/adc.2005.074260
76. Rind E, Pearce J. The spatial distribution of campylobacteriosis in New Zealand, 1997-2005. Epidemiology & Infection. 2010;138(10):1359-71. doi:http://dx.doi.org/10.1017/S095026881000018X
77. Rodrigues LC, Cowden JM, Wheeler JG, Sethi D, Wall PG, Cumberland P, et al. The study of infectious intestinal disease in England: Risk factors for cases of infectious intestinal disease with Campylobacter jejuni infection. Epidemiology and infection. 2001;127(2):185-93; .
78. Sakuma M, Urashima M, Okabe N. Verocytotoxin-producing Escherichia coli, Japan, 1999-2004. Emerging Infectious Diseases. 2006;12(2):323-5.
79. Sargeant JM, Majowicz SE, Snelgrove J. The burden of acute gastrointestinal illness in Ontario, Canada, 2005-2006. Epidemiology and infection. 2008;136(4):451-60. doi:10.1017/S0950268807008837
80. Satterthwaite P, Pritchard K, Floyd D, Law B. A case-control study of Yersinia enterocolitica infections in Auckland. Australian and New Zealand Journal of Public Health. 1999;23(5):482-5; .
81. Scallan E, Fitzgerald M, Collins C, Crowley D, Daly L, Devine M, et al. Acute gastroenteritis in northern Ireland and the Republic of Ireland: a telephone survey. Communicable disease and public health / PHLS. 2004;7(1):61-7.
82. Seo JY, Seo JH, Kim MH, Ki M, Park HS, Choi BY. Pattern of hepatitis A incidence according to area characteristics using national health insurance data. Journal of Preventive Medicine and Public Health. 2012;45(3):164-73; . doi:10.3961/jpmph.2012.45.3.164

83. Seo JY, Choi BY, Ki M, Jang HL, Park HS, Son HJ, et al. Risk factors for acute hepatitis a infection in Korea in 2007 and 2009: A case-control study. Journal of Korean Medical Science. 2013;28(6):908-14. doi:10.3346/ jkms.2013.28.6.908
84. Sethi D, Cumberland P, Hudson MJ, Rodrigues LC, Wheeler JG, Roberts JA, et al. A study of infectious intestinal disease in England: risk factors associated with group A rotavirus in children. Epidemiology and infection. 2001;126(1):63-70.
85. Simonsen J, Frisch M, Ethelberg S. Socioeconomic risk factors for bacterial gastrointestinal infections. Epidemiology. 2008;19(2):282-90. doi:10.1097/EDE.0b013e3181633c19
86. Spencer SE, Marshall J, Pirie R, Campbell D, Baker MG, French NP. The spatial and temporal determinants of campylobacteriosis notifications in New Zealand, 2001-2007. Epidemiology & Infection. 2012;140(9):1663-77. doi:http://dx.doi.org/10.1017/S0950268811002159
87. Stafford RJ, Schluter P, Kirk M, Wilson A, Unicomb L, Ashbolt R, et al. A multi-centre prospective case-control study of campylobacter infection in persons aged 5 years and older in Australia. Epidemiology and infection. 2007;135(6):978-88. doi:10.1017/s0950268806007576
88. Stone DH, Mitchell S., Packham B., Williams J. Prevalence and first-line treatment of diarrhoealsymptoms in the community. Public health. 1994;108(1):61-8; . doi:10.1016/s0033-3506(05)80037-x
89. Tam CC, Viviani L, Rodrigues LC, O'Brien SJ. The second study of infectious intestinal disease (IID2): Increased rates of recurrent diarrhoea in individuals aged 65 years and above. BMC public health. 2013;13(1). doi:10.1186/1471-2458-13-739
90. Teschke K, Bellack N, Shen H, Atwater J, Chu R, Koehoorn M, et al. Water and sewage systems, socio-demographics, and duration of residence associated with endemic intestinal infectious diseases: a cohort study. BMC public health. 2010;10:767. doi:http://dx.doi.org/10.1186/1471-2458-10-767
91. Turkish Ministry of Health. Turkey 1993: results from the demographic and health survey. Studies in family planning. 1995;26(3):180-4.
92. Unicomb LE, Dalton CB, Gilbert GL, Becker NG, Patel MS. Age-specific risk factors for sporadic Campylobacter infection in regional Australia. Foodborne Pathog Dis. 2008;5(1):79-85. doi:10.1089/fpd.2007.0047
93. Van Cauteren D, De Valk H, Vaux S, Le Strat Y, Vaillant V. Burden of acute gastroenteritis and healthcare-seeking behaviour in France: a population-based study. Epidemiology and infection. 2012;140(4):697-705. doi:10.1017/S0950268811000999
94. Varga C, Pearl DL, McEwen SA, Sargeant JM, Pollari F, Guerin MT. Evaluating area-level spatial clustering of Salmonella Enteritidis infections and their socioeconomic determinants in the greater Toronto area, Ontario, Canada (2007 - 2009): a retrospective population-based ecological study. BMC public health. 2013;13:1078. doi:http://dx.doi.org/10.1186/1471-2458-13-1078
95. Weisent J, Rohrbach B, Dunn JR, Odoi A. Socioeconomic determinants of geographic disparities in campylobacteriosis risk: a comparison of global and local modeling approaches. Int J Health Geogr. 2012;11(1):45. doi:10.1186/1476-072X-11-45
96. Whitney BM, Mainero C, Humes E, Hurd S, Niccolai L, Hadler JL. Socioeconomic status and foodborne pathogens in Connecticut, USA, 2000–20111. Emerging Infectious Diseases. 2015;21(9):1617-24. doi:10.3201/eid2109.150277
97. Wilking H, Hohle M, Velasco E, Suckau M, Eckmanns T. Ecological analysis of social risk factors for Rotavirus infections in Berlin, Germany, 2007-2009. Int J Health Geogr. 2012;11:37. doi:http://dx.doi.org/10.1186/1476-072X-11-37
98. Wilking H, Spitznagel H, Werber D, Lange C, Jansen A, Stark K. Acute gastrointestinal illness in adults in Germany: a population-based telephone survey. Epidemiology & Infection. 2013;141(11):2365-75. doi:http://dx.doi.org/10.1017/S0950268813000046

99. Xu Z, Hu W, Tong S. The geographical co-distribution and socio-ecological drivers of childhood pneumonia and diarrhoea in Queensland, Australia. Epidemiology & Infection. 2015;143(5):1096-104.
100. Younus M, Hartwick E, Siddiqi AA, Wilkins M, Davies HD, Rahbar M, et al. The role of neighborhood level socioeconomic characteristics in Salmonella infections in Michigan (1997-2007): assessment using geographic information system. Int J Health Geogr. 2007;6:56.
101. Younus M, Wilkins MJ, Davies HD, Rahbar MH, Funk J, Nguyen C, et al. The role of exposures to animals and other risk factors in sporadic, non-typhoidal Salmonella infections in Michigan children. Zoonoses & Public Health. 2010;57(7-8):e170-6. doi:http://dx.doi.org/10.1111/j.1863-2378.2010.01324.x
102. Zappe Pasturel B, Cruz-Cano R, Rosenberg Goldstein RE, Palmer A, Blythe D, Ryan P, et al. Impact of rurality, broiler operations, and community socioeconomic factors on the risk of campylobacteriosis in Maryland. Am J Public Health. 2013;103(12):2267-75. doi:10.2105/AJPH.2013.301338

Table B3: Excluded studies with rationale

First Author	Year	Reason for exclusion
Admoni	1995	Non OECD at time of data collection
Aksoy	2007	Asymptomatic participants potentially included
Allerberger	1996	No analysis by disadvantaged compared to advantaged
Alter	1982	Outcome not acute-GI infection
Alter	1989	Outcome not acute-GI infection
Alvarado-Esquivel	2014	Asymptomatic participants potentially included
Alvarez-Muñoz	1999	Asymptomatic participants potentially included
Andrews	2003	No analysis by disadvantaged compared to advantaged
Araya	1986	Non OECD at time of data collection
Art	1991	No analysis by disadvantaged compared to advantaged
Baaten	2007	Asymptomatic participants potentially included
Baker	2012	Outcome not acute-GI infection
Bell	2005	Asymptomatic participants potentially included
Bilenko	1999	Non OECD at time of data collection
Blackman	1989	No analysis by disadvantaged compared to advantaged
Bojalil	1994	Non OECD at time of data collection
Bollag	1980	No analysis by disadvantaged compared to advantaged
Bolumar	1995	Asymptomatic participants potentially included
Boreham	1981	Asymptomatic participants potentially included
Bozkurt	2001	No analysis by disadvantaged compared to advantaged
Brandt	1983	No analysis by disadvantaged compared to advantaged
Brieseman	1985	No analysis by disadvantaged compared to advantaged
Brieseman	1990	No analysis by disadvantaged compared to advantaged
Britt	2005	No analysis by disadvantaged compared to advantaged
Broner	2010	Outcome not acute-GI infection
Bryant	1989	No analysis by disadvantaged compared to advantaged
Bura	2012	Asymptomatic participants potentially included
Burström	2005	Data collected pre-1980
Buti	2006	Asymptomatic participants potentially included
Bytzer	2001	Outcome not acute-GI infection
Calderon	1990	Not available in English
Callery	2010	Outcome not acute-GI infection
Cantey	2011	No population comparison
Cedillo-Rivera	2009	Asymptomatic participants potentially included
Çeliksöz	2005a	Asymptomatic participants potentially included
Çeliksöz	2005b	Asymptomatic participants potentially included
Ceran	2012	Asymptomatic participants potentially included
Cevahir	2013	Asymptomatic participants potentially included
Ceyhan	2008	Asymptomatic participants potentially included
Charlett	2003	No analysis by disadvantaged compared to advantaged
Chiaramonte	1983	Asymptomatic participants potentially included
Cifuentes	1999	Outcome not acute-GI infection
Cifuentes	2002	Study participants duplicated elsewhere
Cilla	2010	No analysis by disadvantaged compared to advantaged
Colak	2002	Asymptomatic participants potentially included
Collins	2015	Outcome not acute-GI infection
Connelly	2014	Outcome not acute-GI infection
Conway	1990	Outcome not acute-GI infection
Cross	2009	No analysis by disadvantaged compared to advantaged

Davila	2009	No analysis by disadvantaged compared to advantaged
Davis	2013	No analysis by disadvantaged compared to advantaged
De Wit	2001b	No analysis by disadvantaged compared to advantaged
Deveci	2014	Asymptomatic participants potentially included
Diel	2001	No analysis by disadvantaged compared to advantaged
Ditah	2014	Asymptomatic participants potentially included
Domínguez	2007	Asymptomatic participants potentially included
Doni	2015	No analysis by disadvantaged compared to advantaged
Dostal	2001	No analysis by disadvantaged compared to advantaged
Drobeniuc	2013	No analysis by disadvantaged compared to advantaged
Dubnov	2004	Non OECD at time of data collection
Ekramul Hoque	2002	No analysis by disadvantaged compared to advantaged
Ellencweig	1986	Non OECD at time of data collection
elSaadany	2002	No analysis by disadvantaged compared to advantaged
Erdogan	2012	Asymptomatic participants potentially included
Erdogan	2004	Asymptomatic participants potentially included
Ersoy	1998	Asymptomatic participants potentially included
Escobedo	2011	No analysis by disadvantaged compared to advantaged
Esparza-Aguilar	2014	Outcome not acute-GI infection
Esparza-Aguilar	2013	Outcome not acute-GI infection
Faulkner	2003	Asymptomatic participants potentially included
Feeney	1998	Outcome not acute-GI infection
Fewtrell	1994	Study participants duplicated elsewhere
Finkelman	1994	Non OECD at time of data collection
Ford-Jones	2000	No analysis by disadvantaged compared to advantaged
Forman	1984	No analysis by disadvantaged compared to advantaged
Forsberg	2009	Review/case report/RCT
Francis	1984	No analysis by disadvantaged compared to advantaged
Fraser	1998	Non OECD at time of data collection
Frost	2004	Asymptomatic participants potentially included
Galanis	2014	No population comparison
Gangarosa	1992	Outcome not acute-GI infection
Gastañaduy	2013	No analysis by disadvantaged compared to advantaged
Gibson	1985	Outcome not acute-GI infection
Gomes	2011	Outcome not acute-GI infection
Graham	1988	No analysis by disadvantaged compared to advantaged
Grimwood	2006	No population comparison
Guerrero	2004	No analysis by disadvantaged compared to advantaged
Haley	2010	No analysis by disadvantaged compared to advantaged
Hannah	2005	Outcome not acute-GI infection
Harter	1982	No analysis by disadvantaged compared to advantaged
Hayes-Bautista	1994	No analysis by disadvantaged compared to advantaged
Hemmelgarn	1993	No analysis by disadvantaged compared to advantaged
Hepworth	2010	Study participants duplicated elsewhere
Hizo-Abes	2013	No population comparison
Ho	1988	Non OECD at time of data collection
Hoque	2002	No analysis by disadvantaged compared to advantaged
Howell	2006	Outcome not acute-GI infection
Huerta	2006	No analysis by disadvantaged compared to advantaged
Hughes	2013	Outcome not acute-GI infection
Ikram	1994	No analysis by disadvantaged compared to advantaged
Imhoff	2004	Study participants duplicated elsewhere

Jayasinghe	2013	No analysis by disadvantaged compared to advantaged
Jiménez-Moleón	2011	No analysis by disadvantaged compared to advantaged
Jones	2007	No analysis by disadvantaged compared to advantaged
Jones	1994	Review/case report/RCT
Julio	2012	Asymptomatic participants potentially included
Kanra	2002	Asymptomatic participants potentially included
Kapperud	2003	No analysis by disadvantaged compared to advantaged
Kapperud	1995	No analysis by disadvantaged compared to advantaged
Karaman	2015	No analysis by disadvantaged compared to advantaged
Kaya	2007	Asymptomatic participants potentially included
Koopman	1989	Asymptomatic participants potentially included
Krebs	2011	Non OECD at time of data collection
Kruszon-Moran	2005	Asymptomatic participants potentially included
Kuhls	1994	No analysis by disadvantaged compared to advantaged
Kurugöl	2003	No population comparison
Kurugöl	2011	Asymptomatic participants potentially included
Kurugöl	2009	Asymptomatic participants potentially included
Kyle	2012	Outcome not acute-GI infection
Lazcano-Ponce	2013	Asymptomatic participants potentially included
Leach	1999	Asymptomatic participants potentially included
Leach	2000	Asymptomatic participants potentially included
Lee	2013	No analysis by disadvantaged compared to advantaged
LeJeune	2010	Review/case report/RCT
Lerman	1999	No analysis by disadvantaged compared to advantaged
Leshem	2015	No analysis by disadvantaged compared to advantaged
Letaief	2005	Asymptomatic participants potentially included
Levesque	2013	No analysis by disadvantaged compared to advantaged
Levine	1993	No analysis by disadvantaged compared to advantaged
Levy	1998	No analysis by disadvantaged compared to advantaged
Liddle	1997	No analysis by disadvantaged compared to advantaged
Long	2006	Review/case report/RCT
Lopman	2012	Outcome not acute-GI infection
Lupo	1989	No analysis by disadvantaged compared to advantaged
Ma	2009	No analysis by disadvantaged compared to advantaged
Maguire	1995	No analysis by disadvantaged compared to advantaged
Majowicz	2004	Study participants duplicated elsewhere
Maltezou	2001	No population comparison
Manasek	2004	No analysis by disadvantaged compared to advantaged
Maral	2010	Asymptomatic participants potentially included
Markus	2011	Asymptomatic participants potentially included
Martínez-García	1989	Non OECD at time of data collection
Masia	2004	No analysis by disadvantaged compared to advantaged
McCann	2002	No analysis by disadvantaged compared to advantaged
McQuillan	2004	Asymptomatic participants potentially included
Medeiros	2006	Review/case report/RCT
Mehal	2012	Outcome not acute-GI infection
Mor	2015	Asymptomatic participants potentially included
Morales	1992	Asymptomatic participants potentially included
Morris	1983	Data collected pre-1980
Moyo	2014	Non OECD at time of data collection
Muhsen	2014	Non OECD at time of data collection
Muhsen	2010	Non OECD at time of data collection

Mullner	2010	No analysis by disadvantaged compared to advantaged
Naess	2012	No population comparison
Najnin	2014	No analysis by disadvantaged compared to advantaged
Nathwani	1995	Review/case report/RCT
Nelson	1985	Review/case report/RCT
Newbold	2013	No analysis by disadvantaged compared to advantaged
Newman	1999	No quantitative results
Nicoll	2000	No analysis by disadvantaged compared to advantaged
Noone	2000	No analysis by disadvantaged compared to advantaged
North	1999	No analysis by disadvantaged compared to advantaged
Novotny	1990	Asymptomatic participants potentially included
Ochnio	2005	Asymptomatic participants potentially included
Okur	2011	Not available in English
Okyay	2004	Asymptomatic participants potentially included
Olcay	2003	Asymptomatic participants potentially included
Olsen	2006	No analysis by disadvantaged compared to advantaged
Omurtag	2013	No analysis by disadvantaged compared to advantaged
Ong	2012	No analysis by disadvantaged compared to advantaged
Ostan	2007	Asymptomatic participants potentially included
Ozturk	2004	No analysis by disadvantaged compared to advantaged
Ozturk	1996	Outcome not acute-GI infection
Painter	2015	No analysis by disadvantaged compared to advantaged
Palti	1984	Non OECD at time of data collection
Parashar	1998	Outcome not acute-GI infection
Pasquini	1984	Asymptomatic participants potentially included
Patel	2015	Asymptomatic participants potentially included
Peasey	2004	Asymptomatic participants potentially included
Pérez-Rubio	2011	No analysis by disadvantaged compared to advantaged
Pollock	2006	No analysis by disadvantaged compared to advantaged
Potter	2003	No analysis by disadvantaged compared to advantaged
Psichogiou	1995	No analysis by disadvantaged compared to advantaged
Quihui	2010	Asymptomatic participants potentially included
Quihui	2006	Asymptomatic participants potentially included
Quihui-Cota	2015	Asymptomatic participants potentially included
Quinlan	2013	Review/case report/RCT
Rajan	1998	Asymptomatic participants potentially included
Redlinger	1998	No analysis by disadvantaged compared to advantaged
Redlinger	1997	Asymptomatic participants potentially included
Redlinger	2002	No analysis by disadvantaged compared to advantaged
Rees	1995	No analysis by disadvantaged compared to advantaged
Ricotta	2014	No analysis by disadvantaged compared to advantaged
Rishpon	1984	Non OECD at time of data collection
Roos	2005	Outcome not acute-GI infection
Russo	1997	Outcome not acute-GI infection
Sandberg	2006	No analysis by disadvantaged compared to advantaged
Scavia	2012	No analysis by disadvantaged compared to advantaged
Schmeer	2009	No analysis by disadvantaged compared to advantaged
Sénécal	2008	No population comparison
Sepulveda	1988	Non OECD at time of data collection
Silk	2012	Review/case report/RCT
Snel	2009a	Study participants duplicated elsewhere
Snel	2009b	No analysis by disadvantaged compared to advantaged

Spencer	1988	Non OECD at time of data collection
Stafford	2007	No analysis by disadvantaged compared to advantaged
Standeart	2015	No analysis by disadvantaged compared to advantaged
Stone	1993	No population comparison
Strauss	2001	No analysis by disadvantaged compared to advantaged
Stroffolini	1990	Asymptomatic participants potentially included
Stroffolini	1991	Asymptomatic participants potentially included
Stroffolini	1989	Asymptomatic participants potentially included
Stroffolini	1996	Outcome not acute-GI infection
Studahl	2000	No analysis by disadvantaged compared to advantaged
Talbot-Smith	2002	No analysis by disadvantaged compared to advantaged
Tappero	1995	No analysis by disadvantaged compared to advantaged
Termorshuizen	2000	Asymptomatic participants potentially included
Teshale	2015	Asymptomatic participants potentially included
Thomas	1993	Asymptomatic participants potentially included
Thomas	2008	No analysis by disadvantaged compared to advantaged
Thomas	2011	Non OECD at time of data collection
Thoren	1995	No analysis by disadvantaged compared to advantaged
Thoren	1988	No analysis by disadvantaged compared to advantaged
Thornley	2002	No analysis by disadvantaged compared to advantaged
Thrane	2005	No quantitative results
To	1996	Outcome not acute-GI infection
Tosun	2004	Asymptomatic participants potentially included
Uhlig	2014	No analysis by disadvantaged compared to advantaged
Vancelik	2006	Asymptomatic participants potentially included
Vasquez-Garibay	2015	Asymptomatic participants potentially included
Verma	2014	Non OECD at time of data collection
Vranckx	1990	Data collected pre-1980
Vranckx	1984	Asymptomatic participants potentially included
Vrbova	2012	No analysis by disadvantaged compared to advantaged
Vulcano	2007	Asymptomatic participants potentially included
Warburton	1994	No analysis by disadvantaged compared to advantaged
Withers	2002	No analysis by disadvantaged compared to advantaged
Yapicioglu	2002	Asymptomatic participants potentially included
Yoon	2014	Asymptomatic participants potentially included
Zaidi	2012	No analysis by disadvantaged compared to advantaged
Ziv	2011	Non OECD at time of data collection

Figure A: Harvest plot for risk of GI infection by SES, stratified by age, predominant pathogen transmission route and SES measure

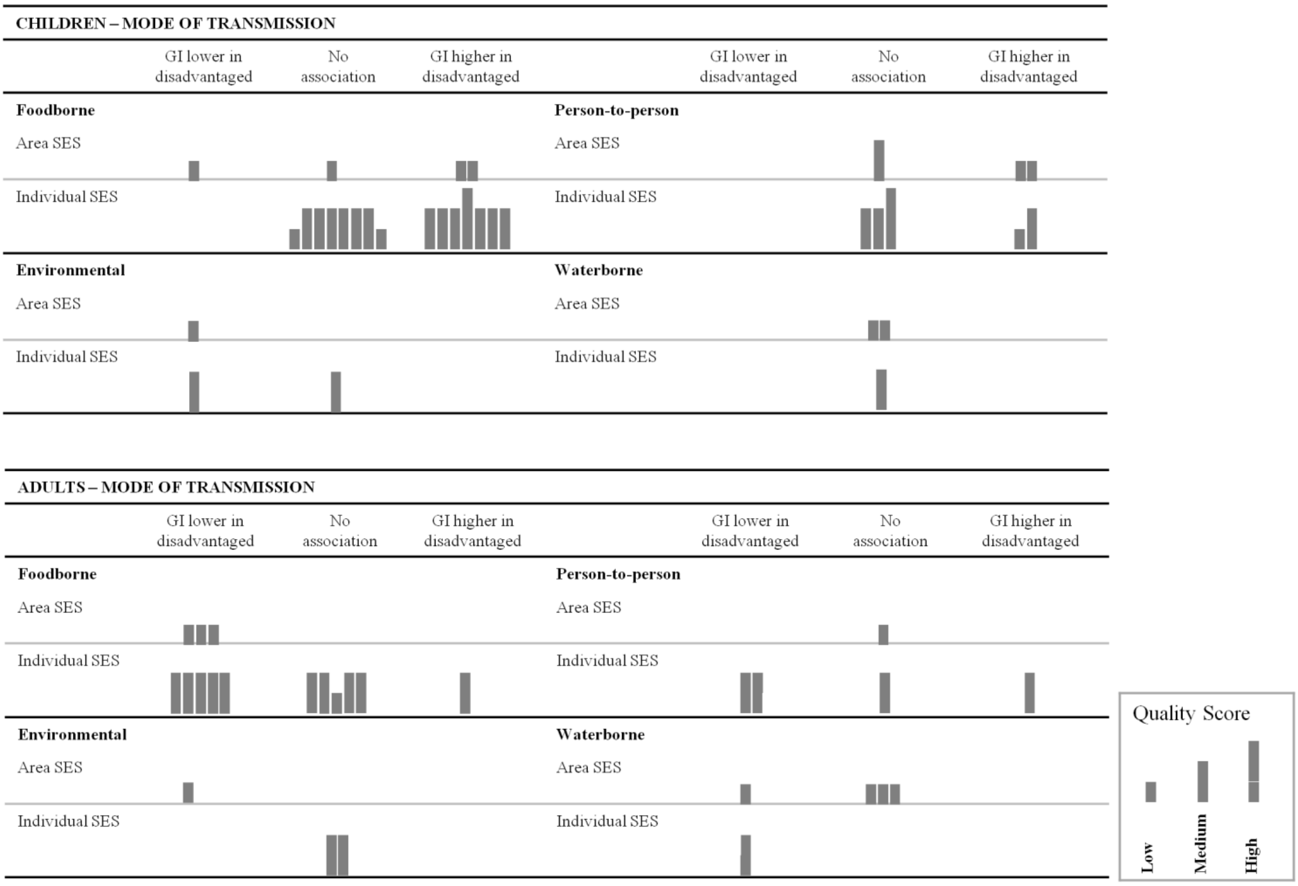

Appendix 3: Legend

Harvest plots were used to demonstrate the relationship between socioeconomic status (SES) and gastrointestinal (GI) infection risk, by displaying and summarising the results of the studies and the subgrouping graphically. Each reported point estimate for the association between SES and GI infection risk was represented by a single bar. The height of the bars were used to indicate the quality score (low, medium or high quality) assigned to the study from which the estimates arose. The process for assigning quality is described in the Methods section. Children were defined as participants of studies aged less than 18 years; adults were defined as participants of studies aged 18 years or older. Estimates were grouped according to the level at which SES was measured, and pathogen type according to the predominant mode of transmission. The harvest plots partition the point estimates into three results: estimates that indicate GI infection risk is lower in more disadvantaged groups; estimates that indicate there is no relationship; and estimates that indicate GI infection risk is higher in more disadvantaged groups.

Figure B: Contour enhanced funnel plot

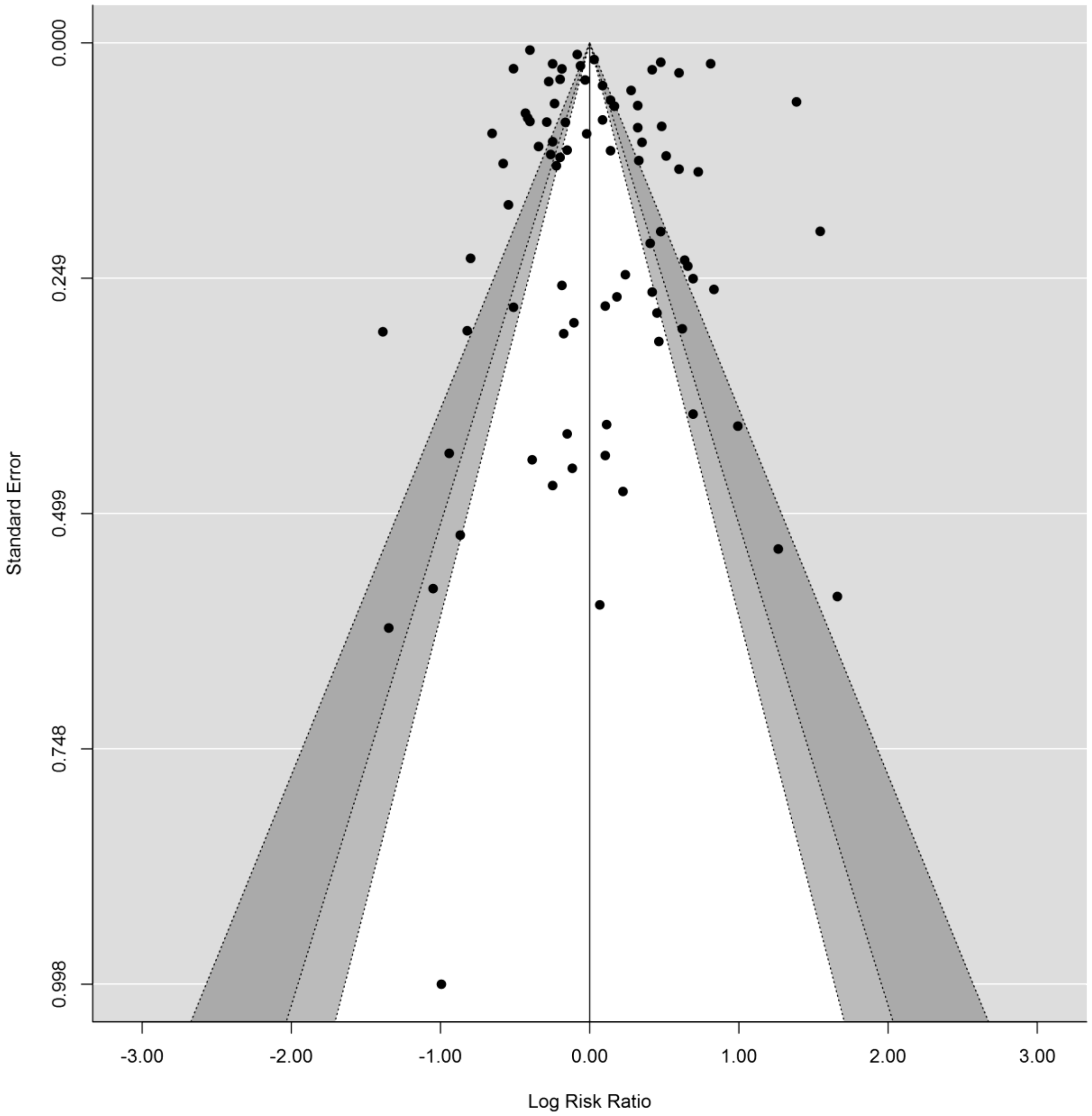

White = P-value > 0.10
Mid-gray = P-value between 0.10 and 0.05
Dark-gray = P-value between 0.05 and 0.01
Region outside of funnel = P-value < 0.01
